# Dietary and homeostatic controls of Zn isotopes in rats: a controlled feeding experiment and modeling approach

**DOI:** 10.1093/mtomcs/mfae026

**Published:** 2024-05-16

**Authors:** Nicolas Bourgon, Théo Tacail, Klervia Jaouen, Jennifer N Leichliter, Jeremy McCormack, Daniela E Winkler, Marcus Clauss, Thomas Tütken

**Affiliations:** IsoTROPIC research group, Max Planck Institute for Geoanthropology, Kahlaische Str. 10, Jena, Germany; Department of Human Evolution, Max Planck Institute for Evolutionary Anthropology, Deutscher Platz 6, Leipzig, Germany; Institute of Geosciences, Johannes Gutenberg University, Johann-Joachim-Becher-Weg 21, Mainz, Germany; Institute of Geosciences, Johannes Gutenberg University, Johann-Joachim-Becher-Weg 21, Mainz, Germany; Department of Human Evolution, Max Planck Institute for Evolutionary Anthropology, Deutscher Platz 6, Leipzig, Germany; Géosciences Environnement Toulouse, Observatoire Midi Pyrénées, 14 avenue Edouard Belin, Toulouse, France; Institute of Geosciences, Johannes Gutenberg University, Johann-Joachim-Becher-Weg 21, Mainz, Germany; HoMeCo Emmy Noether research group, Max Planck Institute for Chemistry, Hahn-Meitner-Weg 1, Mainz, Germany; Department of Human Evolution, Max Planck Institute for Evolutionary Anthropology, Deutscher Platz 6, Leipzig, Germany; Department of Geosciences, Goethe University Frankfurt, Altenhöferallee 1, Frankfurt, Germany; Institute of Geosciences, Johannes Gutenberg University, Johann-Joachim-Becher-Weg 21, Mainz, Germany; Zoology and Functional Morphology of Vertebrates, Zoological Institute, University Kiel, Am Botanischen Garten 3–9, Kiel, Germany; Clinic for Zoo Animals, Exotic Pets and Wildlife, Vetsuisse Faculty, University of Zurich, Winterthurerstr. 260, Zurich, Switzerland; Institute of Geosciences, Johannes Gutenberg University, Johann-Joachim-Becher-Weg 21, Mainz, Germany

**Keywords:** Zinc, stable isotopes, box-model, diet, enamel

## Abstract

The stable isotope composition of zinc (*δ*^66^Zn), which is an essential trace metal for many biological processes in vertebrates, is increasingly used in ecological, archeological, and paleontological studies to assess diet and trophic level discrimination among vertebrates. However, the limited understanding of dietary controls and isotopic fractionation processes on Zn isotope variability in animal tissues and biofluids limits precise dietary reconstructions. The current study systematically investigates the dietary effects on Zn isotope composition in consumers using a combined controlled feeding experiment and box-modeling approach. For this purpose, 21 rats were fed one of seven distinct animal- and plant-based diets and a total of 148 samples including soft and hard tissue, biofluid, and excreta samples of these individuals were measured for *δ*^66^Zn. Relatively constant Zn isotope fractionation is observed across the different dietary groups for each tissue type, implying that diet is the main factor controlling consumer tissue *δ*^66^Zn values, independent of diet composition. Furthermore, a systematic *δ*^66^Zn diet-enamel fractionation is reported for the first time, enabling diet reconstruction based on *δ*^66^Zn values from tooth enamel. In addition, we investigated the dynamics of Zn isotope variability in the body using a box-modeling approach, providing a model of Zn isotope homeostasis and inferring residence times, while also further supporting the hypothesis that *δ*^66^Zn values of vertebrate tissues are primarily determined by that of the diet. Altogether this provides a solid foundation for refined (paleo)dietary reconstruction using Zn isotopes of vertebrate tissues.

## Graphical Abstract

**Graphical Abstract:**
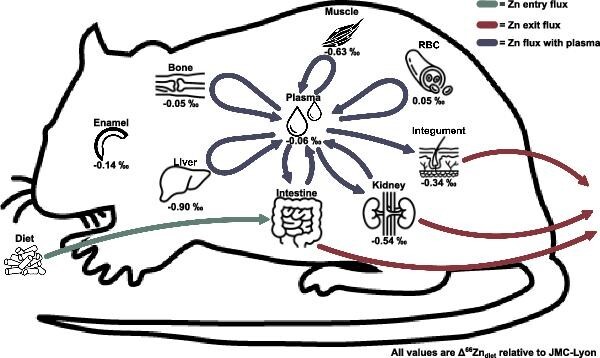
The *δ*^66^Zn values of vertebrate tissues are primarily determined by the diet and the cycling of zinc.

## Introduction

Zinc (Zn) is a trace element with a reactive stability in the cellular environment governed by oxidation–reduction processes through its single oxidation state (2^+^). As the second most abundant micronutrient in the human body, Zn is necessary for living organisms and essential for many biological processes.^1–5^ It is estimated to bind as many as 3000 different proteins in the human body with a wide variety of functions.^6–9^ It binds to a large number of ligands, and it is involved in the activity of over 300 enzymes of all classes and most of the regulatory proteins, as well as many biochemical functions.^1,2^

In order to ensure all Zn-dependent functions and compensate for endogenous Zn losses, Zn has to be continuously supplied by dietary intake.^5,10–12^ Up to a saturation plateau, the amount of absorbed Zn depends on its content in the diet, but also on intestinal bioavailability.^13^ Generally, Zn content in animal products is high compared to most plants.^14,15^ Moreover, some compounds, such as phytate, which is naturally found in many plants, have been shown to severely inhibit intestinal Zn bioavailability.^16,17^ Conversely, dietary proteins appear to be associated with higher Zn uptake, particularly for animal proteins,^18–20^ and even appears to negate the impairing effects of phytate and considerably improve Zn bioavailability.^21,22^

Some controlled feeding experiments and homeostasis modeling studies have already expanded our knowledge of element cycling and fractionation. These studies have provided valuable insights into physiological mechanisms driving variability in the Zn isotope system.^23–27^ In particular, *ab initio* calculations and experimental work showed that differences in Zn coordination environments account for *δ*^66^Zn variability between tissues.^26,28–30^ In proteins, Zn is mostly bound to the amino acids histidine, cysteine, glutamate, and aspartate. In theory, tissues with cysteine-rich proteins (found in liver and kidney) should have lower Zn isotope composition (commonly ^66^Zn/^64^Zn ratio expressed as *δ*^66^Zn value) than tissues with histidine-rich proteins (found in red blood cells and plasma). This is because heavier Zn isotopes tend to concentrate in the higher energy bonds found in histidine, although contrasting results emerged for some tissues across studies.^24–27^ These tissue-specific fractionations are considered to be responsible for trophic level discrimination in Zn stable isotopes, as muscle and many soft tissues (the primary tissues of preys consumed by carnivores) are usually depleted in heavy Zn isotopes relative to the diet.^23–26^ Indeed, Zn isotope ratios have been successfully measured in fossil and modern ecosystems to investigate dietary and trophic behaviors of animals from both terrestrial and marine food webs.^24,26,31–43^ Given that plant material typically has higher *δ*^66^Zn values relative to animal-matter, these studies show that a stepwise depletion in heavy Zn isotopes can be observed along food chains, whereby animals of increasingly higher trophic levels will have progressively lower *δ*^66^Zn value. Thus, *δ*^66^Zn has gained popularity as a proxy for reconstructing diet and trophic ecology in the archeological and fossil record. For instance, recent studies have applied this method to medieval human populations, a Late Pleistocene hunter-gatherer from Southeast Asia, a Neanderthal individual from Gabasa, and even Neogene megalodon sharks.^38,40,42,44^

While studies using Zn as a trophic level tracer have been successful, understanding the behavior of the Zn isotopes within the body is necessary to better interpret variability seen within food webs. Notably, the effect of different diets (e.g. plant- or animal-based) and their associated diet-tissue fractionations have yet to be investigated in a single experimental setting. For example, while the increased Zn uptake and bioavailability associated with animal proteins should presumably favor *δ*^66^Zn values indicative of carnivory for a mixed plant- and meat-based diet, the *δ*^66^Zn values for omnivorous species remain isotopically and statistically distinct from those of carnivores and herbivores.^20,21,39,40^ Thus, the Zn isotope composition of consumers appears primarily dependent on the combined dietary *δ*^66^Zn values (i.e. resulting from a mixing based on *δ*^66^Zn values and Zn content of the ingested resources in the diet).

A deeper understanding of Zn homeostasis, dietary transfer function (i.e. the way dietary isotope compositions are transferred to tissues), and its relevance to the mechanisms of Zn isotope fractionation relative to diet in consumers, is necessary to firmly ground Zn isotopes as a (paleo)dietary proxy. Multiple kinetic models using Zn isotope labelling experiments were previously presented in nutritional studies to establish the fluxes of Zn between compartments (used thereafter to designate tissues, biofluids, and excreta).^45–47^ However, the distributions of the Zn natural stable isotopes were not studied in these models. The present work builds upon previous kinetic models, using *δ*^66^Zn data collected from rats fed a custom-made and controlled diet, thereby enabling the development of dynamic numerical models of Zn isotope variability in each bodily compartment.

Here a Zn isotope dataset is presented from three controlled feeding experiments performed on rats (*Rattus norvegicus* forma domestica). In a first experiment (Experiment-1: Basic Diet), animals received different meat-, insect-, or plant-based pelleted diets (Table S3), in a second experiment (Experiment-2: Bone Addition; Table S3), they received meat-based pelleted diet (the same as in Experiment-1) with a bone-meal supplement used to simulate bone consumption as seen, for instance, in hyenas, while in the third experiment (Experiment-3: Natural diet), animals received (non-pelleted) natural diets (vegetable mix and day-old-chicks).^36,40,44^ This study aims to (1) determine the dependence of mammalian tissues’ *δ*^66^Zn values on the isotopic composition and type of food ingested (e.g. plant-based, animal-based, etc.), and (2) discuss the relationship through box (compartment) models of Zn isotope homeostasis between the isotopic ratios of food products and those of body tissues, biofluids, and excreta. Specifically, the box models can help evaluate and describe turnover times and Zn mass in each tissue and biofluid. Additionally, empirically determined enamel-diet spacings are reported for the first time, representing a crucial step for paleodietary studies.

## Materials and methods

### Controlled feeding experiments design

Controlled feeding experiments described in the present study were performed with ethical approval of the Swiss Cantonal Animal Care and Use Committee in Zurich, Switzerland (animal experiment license no. ZH135/16) at the University of Zurich between July 2017 and March 2018. Experimental setups are described in detail in previous studies.^48–51^ In brief, adult female rats WISTAR (RjHan: WI; 11–14 weeks old at the beginning of the experiment; *n* = 138) were housed in groups of six individuals in separate indoor enclosures.

**Fig. 1:**
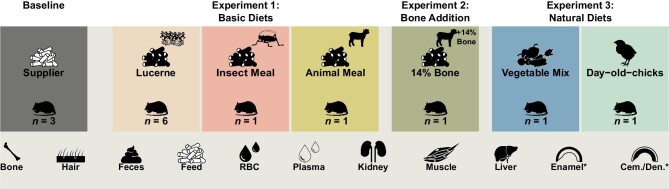
Overview of the experimental setups included in the current study, along with the number of individuals per diet group and samples analyzed (i.e. tissues, biofluids, excreta, and feed). *Denotes that enamel and cementum/dentine (Cem./Den.) samples were only sampled and analyzed for the Basic Diets (Experiment 1) and the supplier's diet (Baseline).

A total of 21 individuals and 3 different experimental setups were used in the framework of the current study (*n* = 148; Fig. 1 and Table 1): (1) Basic Diets (three diets); (2) Bone Addition (one diet), and (3) Natural Diets (two diets). Diets from the Basic Diets Experiment include pelleted animal meal (lamb) diet (*n* = 1), pelleted insect meal diet (*n* = 1), and pelleted lucerne meal (*n* = 6). Diet from the Bone Addition Experiment consists of a pelleted animal meal diet with a bone-meal supplement amounting to 14% of the feed's weight (*n* = 1). Finally, the Natural Diet Experiment includes a vegetable mix diet (*n* = 1) and a day-old-chick diet (*n* = 1). Three rats that received only their respective standard breeder's diet (herein referred to as supplier's diet), were also sampled and used as a group in full isotopic equilibrium. Indeed, the last dietary switch (i.e. weaning from breastmilk) occurred ca. 60 days before the start of this experiment when the rats’ body weights were more than 4 times lower (ca. 50 g at 20 days against 200 g at 80 days). Owing to the fast replacement of the Zn pools during this growth period, we assume that the rats reached diet-body isotope equilibrium by the start of the experiment.

**Table 1:** List and numbers of tissues, biofluids, and excreta samples analyzed in the current study for each experimental setup (Basic Diets, Bone Addition, and Natural Diets) and their respective diet (feed, below), as well as a control supplier's pelleted diet

	**Baseline**	**Experiment-1** **Basic diets**			**Experiment-2** **Bone addition**	**Experiment-3** **Natural diets**		
	**Supplier**	**Lucerne**	Animal meal*	Insect meal*	**14% Bone**	Vegetable mix*	Day-old-chicks*	**Total**
**Bone**	3	6	1	1	1	1	2	**15**
**Enamel**	2	4	5	3	0	0	0	**14**
**Cem./Den.**	2	5	5	3	0	0	0	**15**
**Feces**	3	6	1	1	1	1	1	**14**
**Hair**	1	6	1	1	1	2	1	**13**
**Kidney**	3	6	1	1	1	1	2	**15**
**Liver**	3	6	1	1	1	1	2	**15**
**Muscle**	2	6	1	1	1	1	1	**13**
**Plasma**	1	1	1	1	1	1	1	**7**
**RBC**	3	1	2	2	1	3	1	**13**
**Total**	**23**	**47**	**19**	**15**	**8**	**11**	**11**	**134**
**Feed**	**5**	**3**	**1**	**1**	**1**	**1**	**1**	**13**

Cem./Den. designate the cementum/dentine samples, and RBC the red blood cell ones. In addition to the 147 samples listed above, the bone-meal supplement from the Bone Addition experiment was also analyzed.

*Designates diets for which tissues were fully analyzed for only one individual per diet, but having a few tissues and biofluids from other individuals.

Prior to the experiments (i.e. from birth until arrival in Zurich), all individuals were weaned from mother's milk at ca. 21 days after birth and then received their supplier's diet (Envigo propriety formula 2018S Teklad Global 18% Protein Rodent Diet, for which the main ingredient and primary source of Zn is wheat) until 77–84 days of age, up to 98 days for individuals from the Natural Diet experiment. One group fed only their supplier's diet was kept as baseline, while all others were then fed the supplier diet alongside one of the experimental diets for 5 days to allow acclimatization to the diet. Animals from each group were subsequently fed with one of the experimental foods (54 days for the Basic Diets and Bone Addition experiments, and 32 days for the Natural Diet experiment) and housed under the same conditions. Each enclosure was equipped with two open food dishes containing their assigned experimental diet and two nipple drinkers containing local tap water. Each feeding group received local Zurich tap water. Given the very low Zn concentration in the drinking tap water (ca. 0.005 μg/ml) compared with that of the different feeds (from 24 and 32 μg/g in the vegetable mix and day-old-chicks diets, up to 88 μg/g in the 14% Bone diet), its contribution to daily dietary Zn budget is considered negligible. As the animals received a 15 g daily allowance of food and typically drank 25 ml of water daily, the Zn contribution of drinking tap water is at most 0.3% of daily dietary Zn intake. As such, the Zn isotope composition of drinking water was not measured in this study. Individuals from each group from the Basic Diets and Bone Addition experiments were also kept in isolation in metabolic cages for 4 days after at least 20 days on the experimental diet to measure differences in food intake for each diet and individual fecal collection (≥20 g/individual).

Animals were euthanized using CO_2_ and dissected to sample soft and hard tissues (enzymatically macerated in warm water at 55°C to obtain hard tissues) for isotope analysis. A variety of tissues and biofluids were sampled and analyzed for Zn isotope compositions, including bone, hair, kidney, muscle, liver, red blood cells, plasma, enamel, and cementum/dentine, feces, and the experimental feed of each group. Lower mandibular incisors (enamel and cementum/dentine) and distal tibias were chosen as bioapatite tissues for this study. Except for the supplier's diet individuals, all teeth selected in this study are the same as those used for enamel-bound *δ*^15^N analysis.^51^ Lastly, the cementum/dentine samples taken are predominantly composed of the incisors’ outer hardened layer, composed of cementum, as the inside was mostly hollow.^52,53^

For each diet, all tissues, biofluids, and excreta were analyzed for a single individual with the exception of the pelleted lucerne diet (*n* = 6) and the supplier-feed diet (*n* = 3). In some cases, additional individuals were analyzed per diet for certain tissues and biofluids, especially for dentine, enamel, and red blood cells (Table 1). The diet groups are not necessarily equivalent and serve different purposes: Supplier's diet group serves as Zn isotope equilibrium baseline, lucerne and supplier's diets characterize intra-group *δ*^66^Zn variability, and all other groups primarily assess potential large Zn isotope differences across groups based on the diet's nature. It should also be noted the different diets are artificially designed and do not necessarily reproduce a natural diet composition, trophic spacing or, a ‘closed’ experimental context (i.e. natural ecosystem or experimental context where diets’ ingredients and animals are all grown and raised together), and thus might not follow typical Zn trophic level successions expected from a natural food web (i.e. *δ*^66^Zn_carnivore_ < *δ*^66^Zn_bone-eating carnivore_ and *δ*^66^Zn_omnivore_ < *δ*^66^Zn_herbivore_). Moreover, all pelleted diets contain a substantial proportion of plant-matter (73% for pelleted animal meal, 71% for pelleted insect meal, and 65% for bone-meal diet). As such, no primarily animal-matter-based diets are represented in Experiment-1. All diets and experiments are compared with each other and henceforth considered together in a single dataset for the present study with 148 samples that include 21 individuals and 7 different feeds (including the supplier's diet), as well as the bone-meal supplement for the Bone Addition Experiment.

### Zinc isotope measurement

All perfluoroalkoxy (PFA) vials, polypropylene pipette tips and polypropylene microcentrifuge tubes used were cleaned to minimize Zn contamination. Disposable consumables (polypropylene pipette tips and microcentrifuge tubes) were soaked in 6 M suprapure HCl for 48 h and then in ultrapure Milli-Q water for 24 h. PFA vials were rinsed 3 times in ultrapure Milli-Q water and then soaked for 24 h in ultrapure Milli-Q water again. Vials were then soaked in 3 M suprapure HNO_3_ for 12 h at 80°C, in ultrapure Milli-Q water for 12 h at 80°C and finally in 6 M suprapure HCl for 12 h at 80°C.

Zn purification was performed in Picotrace® metal-free clean lab at the Max Planck Institute for Evolutionary Anthropology in Leipzig, Germany. Throughout the entire study, all solutions were prepared with ultrapure Milli-Q water (18.2 MΩ-cm). Samples were digested using two methods. Bioapatite (bone, tooth enamel, and cementum/dentine) were digested for 1 h at 120°C using 1 ml of suprapure HNO_3_ 65% in PFA screw-cap Savillex beakers. All other samples were instead digested with 10 ml of suprapure HNO_3_ (10 minutes ramp to 100°C, 10 minutes plateau at 100°C, 10 minutes ramp to 180°C, and 10 minutes plateau at 180°C) using a microwave reaction system for sample preparation (Anton Paar Multiwave Pro) at the Institute of Geosciences, University of Mainz, or the German Federal Institute for Materials Research and Testing (*Bundesanstalt für Materialforschung und -prüfung* (BAM)), in Berlin.

After digestion, samples were evaporated and then dissolved in ultrapure HBr 1.5 N. Zn is separated for quantitative recovery in two steps using an ion exchange column chromatography method first described in Jaouen *et al*., 2016 (modified from Moynier *et al*., 2006).^36,54^ Zn was purified in 10 ml hydrophobic interaction columns (Macro-Prep® Methyl HIC) on pre-conditioned 1 ml AG-1 × 8 resin (200–400 dry mesh size, 106–180 μm wet bead size). The resin was then cleaned twice using 5 ml 3% suprapure HNO_3_ followed by 5 ml ultrapure Milli-Q water, and subsequently conditioned with 3 ml 1.5 M HBr. After sample loading, 2 ml ultrapure HBr were added for matrix residue elution, followed by Zn elution with 5 ml suprapure HNO_3_. Following the second column chromatography step, samples were evaporated for 13 h at 100°C and dissolved in 1 ml 3% suprapure HNO_3_. Every preparation batch for column chromatography included at least one National Institute of Standards and Technology Standard Reference Materials (NIST SRM 1400, bone ash) and one chemistry blank in order to assess the quality of the column chromatography purification. The values of the NIST SRM consist of in-house long-term measurements and were determined using the same Zn purification procedure applied to the samples.

Except for enamel and cementum/dentine samples, Zn isotope ratio measurements were made using a Thermo Neptune Multi-collector ICP-MS at the Max Planck Institute for Evolutionary Anthropology (Leipzig, Germany), following the Cu doping protocol to correct for instrumental mass fractionation.^55^ Enamel and cementum/dentine samples were measured at the Géosciences Environnement Toulouse, Observatoire Midi-Pyrénées, using the same protocol on a Thermo Neptune Plus Multi-collector ICP-MS. Zinc isotope ratios are expressed relative to the international standard JMC-Lyon, and isotopic abundances are presented in *δ* (delta) notation expressed as deviation per mil (‰), as follows: *δ*^66^Zn = (^66^Zn/^64^Zn sample/^66^Zn/^64^Zn standard – 1) × 1000. The in-house standard Zn AA-MPI (using Alfa Aesar® ICP-MS Zn standard solution) was used for standard bracketing, with mass-dependent offset to JMC-Lyon standard material of +0.27 ‰ for *δ*^66^Zn.^36,56,57^ Analyzed sample solution Zn concentrations were close to 300 ng/g, as was the Zn concentration used for the standard mixture solution. Following a protocol adapted from Copeland for Sr and first used for Zn by Jaouen *et al*. (2016), regression equations based on the ^64^Zn signal intensity (V) of three solutions with known concentrations (150, 300, and 600 ng/g) were used to estimate the Zn concentrations of samples.^36,58^ A reference material NIST SRM 1400 was prepared and analyzed alongside the samples for each column chromatography batch, and had *δ*^66^Zn values (+0.96 ± 0.03 ‰ (1σ), *n* = 13) as reported elsewhere.^40,42^ The *δ*^66^Zn measurement uncertainties were estimated from standard and sample replicate analyses and ranged between ± 0.01 ‰ and ± 0.02 ‰ (1 σ). The reference materials and samples exhibit a Zn mass-dependent isotope fractionation and the absence of isobaric interferences, whereby *δ*^66^Zn vs. *δ*^68^Zn values fall onto lines with slopes close to the respective theoretical mass fractionation values of 2.

### Zn isotope dynamic homeostasis box models

Published Zn kinetic models for rats have mostly focused on evaluating and describing turnover rates and Zn mass in each tissue and biofluid independently, specifically regarding exchanges with the plasma.^45,46^ Here, we first built a reference whole-body model constrained by the isotopic observations in the rats fed the supplier's pelleted diet and assumed to be in isotopic equilibrium with their dietary Zn intake. The time required for organisms to fully equilibrate their tissues’ isotopic compositions with their diet is also a critical consideration that will be assessed, especially considering that the Basic Diets and Bone Addition experiments (Experiments 1 and 2) lasted 54 days, while the Natural Diet experiment (Experiment 3) lasted 32 days. We then proceeded to evaluate the systems through time to a change of diet isotopic composition (Supplementary Material 3). Box models in the current study use mathematical formalism and equations developed elsewhere and ‘isobxr’, an R package designed to investigate stable isotope box modeling of any open or closed system in steady state or in response to a perturbation and first used in.^23,59,60^

We included the 11 following compartments (Table 2): diet (D), intestine (Int), plasma (Pla), erythrocytes (i.e. red blood cells; RBC), liver (Liv), muscles (Msc), bones (Bne), kidneys (Kdn), hair (considered here as integument; Integ), urine (Ur), and feces (Fec). The fluxes (i.e. exchanges) among compartments (in µg/d) were treated as first-order kinetic coefficients or, equivalently, as exit probabilities per unit of time. Excretion (i.e. feces, urine, and desquamation) was also treated as a probability of irreversible loss (i.e. intestine to feces, integument to desquamation, and kidney to urine). While kidneys were prepared and measured for *δ*^66^Zn values in the current study, their fit was excluded for modeling purposes as they are rather used as an interface, and overall too complex to be modeled in this article. Similarly, enamel was excluded altogether as it requires the consideration of additional processes (e.g. enamel secretion and maturation, tooth geometry or sampling resolution, etc.), which are far too complex to be accommodated by the current modeling approach.^61^

**Table 2:** List of Zn masses (μg) in each compartment used in the current study for a rat of 370 g. All masses are taken from House & Wastney (1997) and all δ^66^Zn (‰ JMC-Lyon) using the rats fed supplier's feed from the current study^46^

**Compartment**	**Zn content in compartment (μg)**	***δ* ^66^Zn (‰)**	**Obs.CI**
Diet	Infinite	0.42	0.08
Intestine	1000	–	–
Plasma	32	0.36	0.20
Liver	378	−0.48	0.12
Red Blood Cells	320	0.47	0.03
Muscle	1960	−0.21	0.21
Bone	3212	0.37	0.12
Kidney	63	−0.12	0.12
Integument	2787	0.08	0.20
Feces	813	0.44	0.14
Urine	7	–	–
Waste	Infinite	–	–

The observed confidence intervals (Obs.CI) were defined as 2SE and, in the case of boxes with a single observation (PLA and INTEG), the Obs.CI were conservatively set to 0.2 ‰, corresponding to 4*maximized external reproducibility (roughly 0.05 ‰ on repeated measurement of standards) and also to the maximum of estimated 2SE on other boxes with n ≥ 2 individuals.

Zinc fluxes are taken from House and Wastney (1997), and most Zn isotope fractionation coefficients (α_i__–__j_) were calculated from *δ*^66^Zn values of compartments of rat individuals fed only the supplier's diet taken as a reference of an organism at diet-body isotope equilibrium.^46^ In our modeling approach, we also assume a first-order physiological steady state of the organism (i.e. constant and balanced fluxes as well as constant box sizes and fractionation factors). We therefore do not model growth nor ageing of the adult organism.

While most parameters can be retrieved from the literature or directly assessed from our observations, the nominal values of some Zn fluxes and Zn isotope fractionations in rats remain unknown or poorly constrained. We therefore simulated Zn isotope cycles in rats considering varying values of such fluxes and fractionation factors by sweeping the space of parameters using the sweep.final_nD function of the isobxr R package.^60^ We were then able to estimate the best sets of parameter values allowing to reproduce the observed steady-state Zn isotope compositions (within confidence intervals) reported for the key compartments of the supplier rats organism (Supplementary Material 2) using the fit.final_space function of the isobxr R package. Varying flux configurations were thus swept to encompass the various Zn cycles in mammals, as reported in humans and rats, and fractionation coefficients (using a split fractionation amplitude respectively attributed to efflux and influx) to explain the constant fractionation factors set on Zn influx and efflux, mostly to dead-end soft tissue reservoirs but also for bone. For a set of key compartments, this method permits determination of all combinations of parameter values, which, in turn, allows for exploration and production of steady-state isotope compositions falling within the observed confidence intervals for all compartments of interest (values obtained from rat individuals in equilibrium with their diets). Parameters explored with this method are the following: fractionation upon intestinal absorption (α_INT-PLA_) and endogenous losses (α_PLA-INT_), fractionation upon integument transport (α_PLA-INTEG_), fractionation upon urinary losses (α_KDN-UR_), and fluxes between plasma and integument as well as plasma and bone (i.e. varying residence times).

The resulting Zn cycle, masses, isotope fractionation coefficients, and fluxes between all compartments listed above are presented in Tables 2 and 3.

**Table 3:** List of fluxes (μg/day) and fractionation coefficients between compartments used in the current study for a rat of 370 g

**From (i)**	**To (j)**	**Fluxes (μg/day)**	**Fractionation coefficient (α_i__–__j_)**	**Corresponding Δ_i__–__j_ (‰)**
D	Int	1000^a^	1.00000	0.00
Int	Fec	989	1.00000	0.00
Int	Pla	305	0.99970–1.00030	−0.30 to 0.30
Pla	Int	294	0.99970–1.00030	−0.30 to 0.30
Pla	Liv	900	0.99958	−0.42
Pla	RBC	35	1.00005	0.05
Pla	Msc	227	0.99972	−0.28
Pla	Bne	1.1–231.1	1.00001	0.01
Pla	Integ	4–180	0.99950– 1.00000	−0.50 to 0.00
Pla	Kdn	118	1.00000	0.00
Liv	Pla	900	1.00042	0.42
RBC	Pla	35	0.99995	−0.05
Msc	Pla	900	1.00028	0.28
Bne	Pla	1.1–231.1	0.99999	−0.01
Knd	Pla	111	1.00000	0.00
Kdn	Ur	7	1.0000 and 1.0010	0.00 to 1.00
Ur	Waste	7	1.00000	0.00
Integ	Waste	4	1.00000	0.00
Fec	Waste	989	1.00000	0.00

All fluxes are taken, or modified (^a^), from House & Wastney (1997).^46^ Fractionation coefficients are calculated from δ^66^Zn values from the current study or deduced from the sweep space.

## Results

### Blanks, reproducibility, and precision

The average sample solution Zn content from the chemistry blanks ranged from 0.4 to 6.8 ng (average = 2.3 ± 2.1 ng (1 σ), *n* = 14). As the average Zn content for samples (average sample Zn content = 2454 ± 2548 ng, *n* = 139) is about 1000 times higher, the isotopic composition measured for each sample and reference material is highly unlikely to have been influenced by the blanks, as the potential Zn contribution is too low (i.e. contributing only about 0.09% of the sample solution Zn content). Repeated analyses of some specimens (*n* = 65) and reference material (*n* = 13) were performed to determine the homogeneity of samples, and the overall average analytical repeatability for samples and reference material was ± 0.01 ‰ (1 σ).

### Variation of *δ*^66^Zn values between tissues and diets

**Fig. 2:**
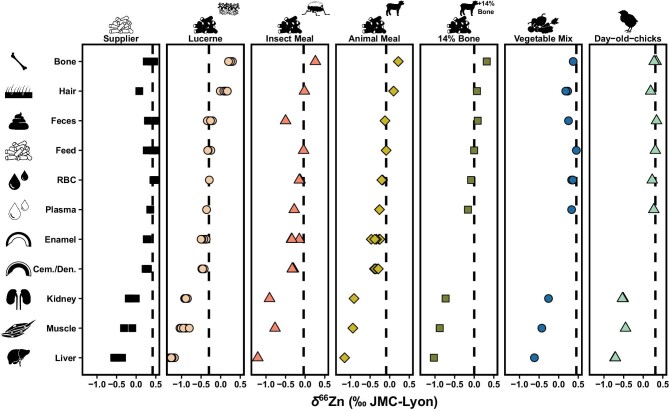
The Zn isotope compositions (‰, relative to the JMC-Lyon Zn isotope standard) of the liver, muscle, kidney, plasma, red blood cells (RBC), feces, hair, bone, enamel, cementum/dentine (Cem./Den.), and respective feeds of animals highlight the generally low δ^66^Zn values in soft tissues and higher values in bones compared with the diet. Each facet, and its corresponding point shapes and colors, are associated with a distinct diet: pelleted supplier's diet, pelleted animal meal diet, pelleted insect meal diet, pelleted lucerne meal, pelleted animal meal diet with a 14% bone-meal supplement, vegetable mix diet, and a day-old-chick diet. The dashed lines correspond to the mean δ^66^Zn values of the diet supplied to the animals.

All results are given in Table S1. The *δ*^66^Zn values recorded in all individuals differed between diets and followed a similar pattern to the Zn isotope compositions of their respective feeds (Fig. 2), with the exception of bone, which has a slow remodeling rate, and hair, which grows in cycles rather than gradually remodeling (see Supplementary Material 2).^62,63^

Inter-individual variation of *δ*^66^Zn values within diet groups was explored in rats for the pelleted lucerne diet and pelleted supplier's diet, whereby typical variation observed for each tissue from each diet (respectively 0.05 ± 0.02 ‰ (1σ) and 0.09 ± 0.05 ‰ (1σ)) is similar to that of the feeds themselves (± 0.04 ‰ (1 σ), *n *= 3; and 0.09 ‰ (1 σ), *n *= 5). For both diets, the *δ*^66^Zn inter-individual variation was higher in muscle tissue than in others (± 0.09 ‰ (1 σ), *n* = 6; and 0.15 ‰ (1 σ), *n* = 2; for the pelleted lucerne diet and pelleted supplier's diet, respectively).

The red blood cells and feces’ *δ*^66^Zn values are close to that of the diet (respectively: Δ^66^Zn_RBC-diet_ = −0.05 ± 0.07 ‰ (1 σ), *n* = 13; and Δ^66^Zn_feces-diet_ = −0.03 ± 0.15 ‰ (1 σ), *n* = 14), whereas plasma exhibits a slight depletion in ^66^Zn relative to the diet (Δ^66^Zn_plasma-diet_ = −0.12 ± 0.07 ‰ (1 σ), *n* = 7). Kidney, muscle, and liver show depletion in ^66^Zn relative to the diet (respectively: Δ^66^Zn_kidney-diet_ = −0.66 ± 0.13 ‰ (1 σ), *n* = 15; Δ^66^Zn_muscle-diet_ = −0.70 ± 0.13 ‰ (1 σ), *n* = 13; and Δ^66^Zn_liver-diet_ = −0.99 ± 0.09 ‰ (1 σ), *n* = 15). Because of the long residence time in bones and hair, only the diet-to-tissue fractionation of animals that did not experience an experimental diet switch (i.e. supplier rats), is considered since those from other diets are not in isotopic equilibrium and still exhibit similar *δ*^66^Zn values to those of individuals only fed the supplier's diet (i.e. they mostly retain a pre-experimental diet value). Both tissues show a depletion in ^66^Zn relative to the diet, whereby the bones are closer to the diet's *δ*^66^Zn value (Δ^66^Zn_bone-diet_ = −0.05 ± 0.10 ‰ (1 σ), *n* = 3), and the hairs are more depleted (Δ^66^Zn_hair-diet_ = −0.34 ‰, *n* = 1).

Individuals fed the lucerne pelleted diet recorded the lowest *δ*^66^Zn values. The animal-based diets (pelleted animal meal, pelleted insect meal, pelleted bone addition meal, and day-old-chick natural diet, respectively following this order of increasing *δ*^66^Zn values) followed, with the supplier's pelleted diet and the natural vegetable mix diet having the highest values. Accordingly, the pelleted bone addition meal (*δ*^66^Zn = 0.00 ‰) showed a shift toward the values of the bone-meal supplement (*δ*^66^Zn = 0.96 ‰) away from the pelleted animal meal (*δ*^66^Zn = −0.09 ‰), with which it was supplemented.

## Discussion

### Evolution of *δ*^66^Zn in a rat body

The sweep CI-fits (Supplementary Material 2) allowed for exploring and establishing sets of parameters, both fluxes and fractionation coefficients, that enabled reproducing the values observed in rats at Zn isotope equilibrium with their diets. While different suites of suitable configurations were identified, this modeling approach also notably highlighted features regarding the steady-state isotope compositions of the organism itself.

In vertebrates, the diet's Zn isotope composition is expected to be the primary control on those of animal tissues. First, we thus explore a Zn isotope homeostasis that assumes animal tissues are isotopically equilibrated with their experimental diets, whereby rat individuals fed only the supplier's diet are used as a steady-state reference (i.e. at diet-body isotope equilibrium and at physiological steady-state). When describing the relationship between the isotopic composition of given tissues and dietary intake or the evolution of a system, a crucial consideration is the time required for organisms to fully equilibrate the isotopic compositions of their tissues with that of their diet. This can be accounted for using a box modeling approach with sufficient knowledge on the typical cycle of a given element (masses and fluxes in and between all compartments) and of the organ in question, whereby the characteristic relaxation times of the exponential solutions of the system of differential equations describing the evolution of the system can be assessed.^23,64,65^ Typically, the system can be considered fully equilibrated with the dietary source within 5 times the longest relaxation time.

**Fig. 3:**
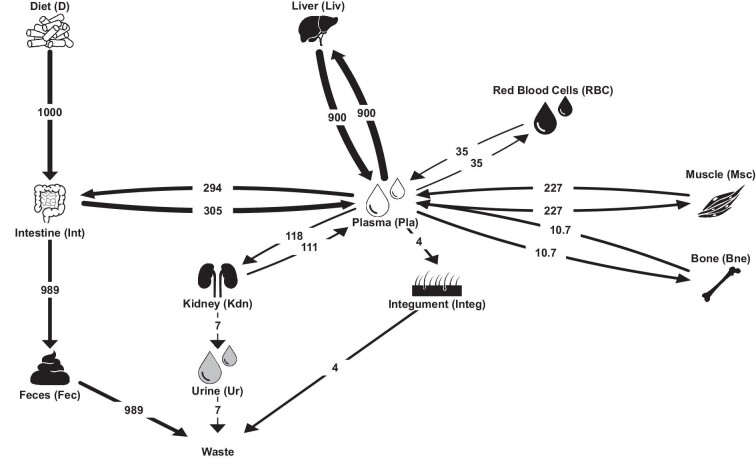
Schematic diagram of the Zn isotope cycle in the body of a rat, in µg/day. For the fractionation coefficients, see Table 3.

Among others, the extent of the renal isotope fractionation appears to have no significant impact overall on bodily baseline *δ*^66^Zn values (Figure S2). Therefore, we decided to set it to 0.44 ‰ (α_KDN-UR _= 1.00044), which is comparable to the average offset in *δ*^66^Zn between urine and plasma previously reported in humans.^66^ The fluxes of Zn from plasma to bone and from bone to plasma were also explored, whereby the residence time of Zn in bone of a first-order physiological steady-state was made to vary between 14 days (as suggested by House and Wastney, 1997) up to ca. 3000 days as upper-end extreme, as assumed if Zn has the same residence time as Ca in humans (e.g. 2000 days if 1 kg Ca in bone and 500 mg/d exchanged).^46,67^ The resulting simulations calibrated against a switch from supplier to Lucerne diet provided a best fit with a t_1/2_ in bones of 300 days (Figure S7), indicating that bones of individuals fed on the experimental diets for 60 days or less do not reflect the isotopic composition of their respective feeds. Our observations and simulations thus support that the Zn residence time in rat bone is at least one order of magnitude longer than the previously suggested 14 days (Supplementary Material 2). Additionally, although dependent on the uncertainty of bone *δ*^66^Zn values, our 300 days estimate appears to be one order of magnitude lower than the expected 2000–3000 days residence time of Ca in bone in humans. This order of magnitude of 300 days is in good agreement with the residence time of Ca in bones of rats, estimated to vary between 250 and 1600 days.^68^ Such differences probably relate to distinct bone remodeling rates between rats and humans, probably owing to the allometry of bone turnover rates in mammals.^69^ Similarly, the best modeled fit (i.e. the one reproducing the *δ*^66^Zn values obtained supplier-fed rat individuals at isotopic equilibrium with their diets) for integument flux (hair in the current case) corresponds to a low integument loss/high endogenous loss ratio (Figure S5). This configuration is comparable to what is reported for humans but different than what was reported for rats by House and Wastney (1997).^46^ This consequently leads to a much longer modeled t_1/2_ of Zn in the integument, of the order of 700 days rather than 70 days, and thus a slower equilibration rate for hair. Therefore, hairs of individuals fed on the experimental diets do not reflect the isotopic composition of their respective feeds on such small timescales. However, it is worth mentioning that the modeling for hair is mostly done to account for integumentary Zn losses and satisfy the rest of the box-model. The current modeling approach assumes a steady hair growth and thus does not accommodate the complexity of rats’ hair growth, which not only follows cycles of roughly 35 days but also retains hairs from previous ones.^62,63^ This also explains the discrepancy between the integument flux observed by House and Wastney (1997) and the best modeled fit obtained in the current study. Nonetheless, although longer than a rat's average lifespan (i.e. 1.5‒2 and up-to but rarely 3 years), the calculated t_1/2_ for bone and hair are not intrinsically unrealistic. If verified, this would simply mean that an adult individual does not fully reach steady-state equilibration between diet and tissues over its life after having been introduced to a new diet. We thereafter used these updated fluxes (plasma to bone, bone to plasma, and plasma to integument) and isotopic fractionation (α_KDN-UR_) for the final configuration used for subsequent modeling (Fig. 3).

**Fig. 4:**
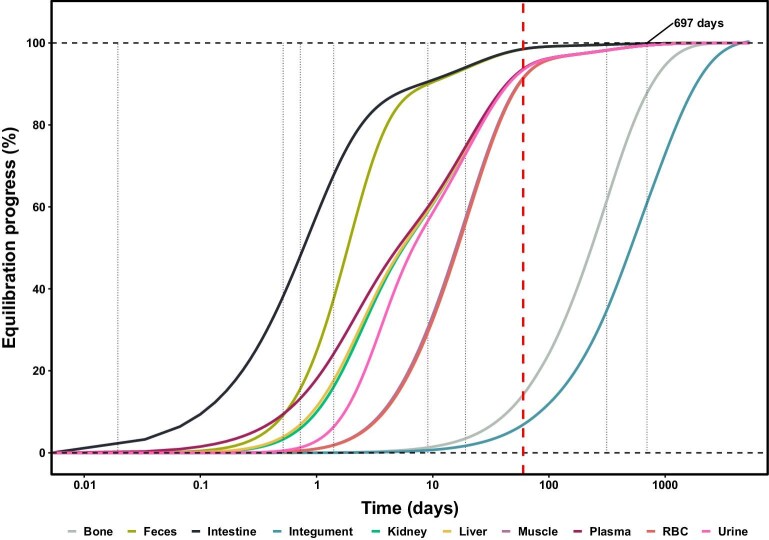
Progress (%) of body-diet Zn isotope equilibration in rats with time (days), assuming an individual that: (1) is an adult animal, (2) is not growing, (3) has no prior dietary switch (such as weaning, for example), and (4) has a typical life expectancy of 730–1095 days. The vertical red dotted lined correspond to the duration of the longest experiments (roughly 60 days for both Experiment-1 and 2), and the characteristic relaxation times (see Table 4) are shown as dotted vertical lines, whereby the maximal one (697 days) is derived from hair Zn residence time.

The relaxation times of the whole system were modeled and demonstrate that almost every tissues and biofluids’ from individuals of Experiment-1 and 2 would be near isotopic equilibrium (∼90% or more; Table 4) with their diet by the end of the experiment's duration (ca. 60 days), except for hair and bone, both with much longer Zn residence times (Fig. 4).

**Table 4:** Residence times t_1/2_ (days) and time to *x*% equilibration progress for each compartment (tissue, excreta, and biofluid) of rats, as well as the whole system relaxation times

	**Residence times**	**Time to *x* % equilibration progress (days)**			Whole system relaxation times (days)*
**Compartment**	**t_1/2_ (days)**	**t_50%_**	**t_95%_**	**t_99%_**	**t_relax_ (sorted)**
Intestine	0.8	1	23	83	0.02
Feces (day loss)	1.0	2	24	84	0.5
Plasma	0.0	5	73	486	0.7
Liver	0.4	5	73	487	1.0
Kidney	0.5	6	73	487	1.0
Urine (day loss)	1.0	7	74	488	1.4
Muscle	8.6	16	84	495	9.1
Red blood cells	9.1	16	85	496	19.1
Bone	300.2	231	954	1460	314.4
Integument	696.8	510	2124	3246	696.8

*Relaxation times are not a direct characteristic of a reservoir but are characteristic of the dynamic behavior of the whole system.

**Fig. 5:**
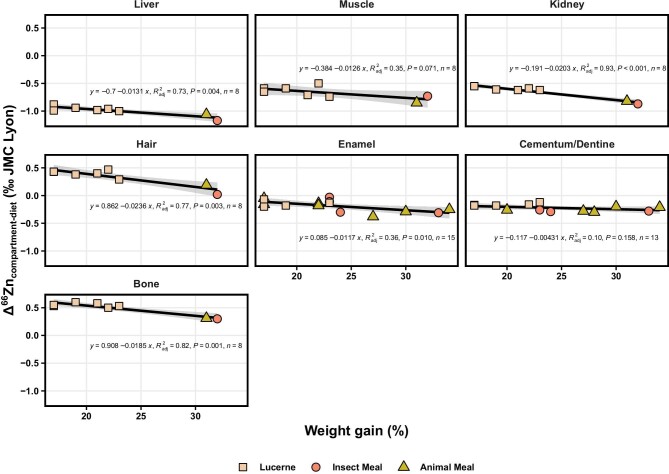
The Zn isotope compositions of different tissues relative to the consumer's diet (Δ^66^Zn values in ‰, relative to the JMC-Lyon Zn isotope standard, whereby the diet is 0.0 ‰) and in relation to body mass gain for individuals of Experiment-1. The change in body mass is expressed as a ratio between the body mass recorded at termination of the experiment (59 days) and the initial body masses before receiving the experimental diet. The black line represents the regression line with 95% confidence interval (standard error as shaded areas). Shapes and colors correspond to different diets from Experiment-1: pelleted lucerne diet, pelleted insect meal diet, and pelleted animal meal diet.

As individuals in the current study were growing, most feeding groups displayed a significant increase in body mass throughout all three experiments (Table S2), with significant differences in the growth performances of animals observed between diets. Within Experiment-1, rats that received the plant-based diet gained 20 ± 6% body mass (*n* = 6), while individuals from the meat and insect groups displayed slightly higher body mass gains, 27 ± 6% and 26 ± 5% (*n* = 6 and *n* *=* 3), respectively. While dietary Zn intake constitutes the primary control over isotopic compositions of the animal tissues, growth performance also appears to have an effect to some degree and seems to generally lead to lower Δ^66^Zn values (Fig. 5).

Indeed, while the time required to fully equilibrate all of the main Zn reservoirs of the organism with dietary Zn exceeds 1000 days (usually 5 times the maximal relaxation time of 697 days), the equilibration is seemingly accelerated by better growth performances, bringing tissues closer to isotopic equilibrium with their dietary source more quickly. Although growth performances seemingly affect *δ*^66^Zn values in tissues, the current models do not take it into account as it would require a more in-depth understanding on precise location of fractionation, including in dead-end reservoirs. Nonetheless, it is worth noting that it can thus perhaps partly explain some differences between individuals and diets between predicted and observed values.

**Fig. 6:**
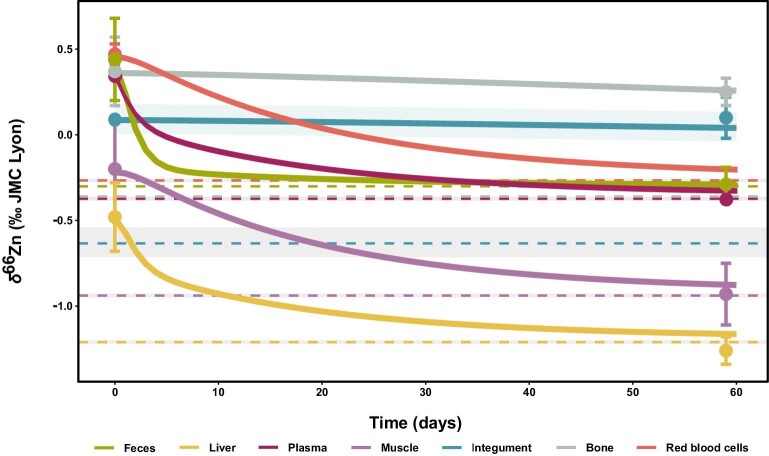
Predicted evolution of δ^66^Zn_diet_ (‰ JMC-Lyon) in each box following a dietary transition from a steady-state organism at equilibrium with the supplier diet to a lucerne pelleted diet. The dashed lines with a shaded area correspond to the predicted steady-state isotope composition when the organism is fully equilibrated with the second diet (lucerne pellets here). The circles correspond to the average values of the observed δ^66^Zn_diet_ values (error bars are 2σ). The time axis corresponds to the time (in days) that elapsed since the start of the experiment (i.e. the introduction of the second diet). Each curve and shaded area represent the average and full extent of compositions predicted by the sets of fitted parameter values determined (see fit_4 described in Supplementary Material 2).

The predictions for the diet-switch modeling (Fig. 6 and Supplementary Material 3) are overall in very good agreement with observed compositions, even though our model does not take ongoing growth nor ageing into account. It is also important to note that not all diet groups are equivalent, as the number of individuals is variable from one group to another, most being represented by a single individual. In contrast, six individuals were analyzed from the lucerne pelleted-feed diet, making it the best suited to evaluate the effect of a diet shift through time as a function of the *δ*^66^Zn value of dietary intake. For some diets, some discrepancies between predicted and observed values, notably for liver, muscle, and RBC, can likely be associated with these differences in the numbers of individuals analyzed, as well as growth performance.

### Natural distribution of Zn isotopes in the rat body

**Fig. 7:**
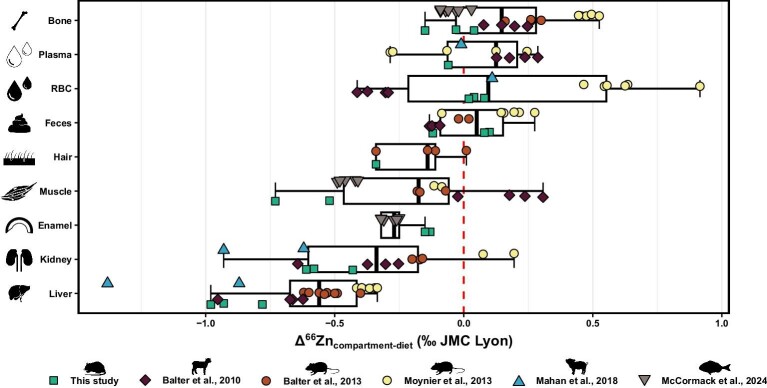
The Zn isotope compositions (‰, relative to the JMC-Lyon Zn isotope standard) relative to a consumer's diet (Δ^66^Zn values) of tissues, biofluids, and excreta found in different controlled feeding experiments and the current study: bone, plasma, red blood cells, feces, hair, muscle, enamel, kidney, and liver. Balter *et al*. (2010) and Moynier *et al*. (2013) measured serum (plasma without clotting agents), and McCormack *et al*. (2024) measured enameloid.^24,26,70^ The red dashed line corresponds to diet's values normalized to 0 ‰, and the symbol shapes and colors correspond to different studies: rats (this study), sheep (Balter *et al*., 2010), mice (Balter *et al*., 2013), circle for mice (Moynier *et al*., 2013), minipig (Mahan *et al*., 2018), and sea breams (McCormack *et al*., 2024).^24–27,70^ For each study, only the specimens that were on their respective experimental diet for the longest time, were selected: 12 weeks (supplier's diet group, this study), 14 and 16 weeks (Moynier *et al.*, 2013), 22 weeks (Balter *et al*., 2013), 52 weeks (Balter *et al*., 2010), 80 weeks (Mahan *et al*., 2018), and 52–69 weeks (McCormack *et al*., 2024).^24–27,70^ The boxes represent the 25th–75th percentiles, with the median represented by a bold horizontal line.

The *δ*^66^Zn values observed for the different compartments are mostly in line with published data, but some notable exceptions can be discerned (Fig. 7), including bone, muscle, and blood (plasma and red blood cells).^24–27,70^ Deviations in *δ*^66^Zn values from literature data could have serious implications for (paleo)dietary reconstructions; bone is one of the preferred archives in paleodietary studies, muscles are the primary tissue consumed by carnivores, and plasma (and red blood cells, as the primary location of Zn in the blood) controls the Zn isotope composition of other compartments.

The present *δ*^66^Zn data from bone diverge somewhat slightly from assumptions of Zn isotope fractionation according to the nature of the metal bonds with ligands, namely regarding Zn bonding in bioapatite. Because heavier Zn preferentially binds to ligands with a stronger electronegativity (O > N > S), enrichment of heavy Zn isotopes is expected in bioapatite due to bonding with oxygen atoms of one hydroxyl (OH) and three phosphate groups (PO_4_), while ^66^Zn depletion is expected in muscle proteins and other soft tissues because of Zn binding to N of various amino acids.^25,26,29,30,71,72^ However, while bioapatite tissues in the current study exhibit enrichment of heavy Zn relative to soft tissues, they nonetheless have similar or lower *δ*^66^Zn values than the diet and plasma. This was also observed in sea breams (*Sparus aurata*) from a pisciculture farm where the reported offsets between bone and diet *δ*^66^Zn values (Δ^66^Zn_bone-diet_ = −0.04 ± 0.04 ‰, (1 σ), *n* = 7) are similar to the current study (Δ^66^Zn_bone-diet_ = −0.05 ± 0.10 ‰ (1 σ), *n* = 3).^70^ Variability in the isotopic equilibrium of the tissue with the diet could be expected to be the main factor in differences with the other studies, but the data are equally inconsistent in supporting this assumption. Indeed, even when only comparing older specimens (i.e. those whose body's *δ*^66^Zn values are at or closest to isotopic equilibrium with their diet: 12 week-old rats (supplier's diet group, this study), 14 and 16 week-old mice, 22 week-old mice, 52 week-old sheep, and 80 week-old minipigs), Δ^66^Zn_bone-diet_ values are different between these studies and the current results presented.^24–27^ Differences in growth performance, Zn bioavailability of the diet, and time since the last dietary switch could all be mentioned as possibilities to explain this discrepancy between these studies.

Differences in Δ^66^Zn_plasma-diet_ and Δ^66^Zn_RBC-diet_ values across studies are also somewhat confounding (Fig. 7). Blood, and its plasma and RBC, is a biofluid with a short residence time (i.e. a fast turnover) and should thus be undoubtedly at isotopic equilibrium with the diet, as also predicted by the box-model approach. While both Balter *et al*. (2010) and Moynier *et al*. (2013) measured serum (plasma without clotting agents), the influence of clotting agents on Zn elemental abundances and isotope composition should be negligible and serum should consequently yield comparable *δ*^66^Zn values with plasma.^73,74^ Differences with these studies in Δ^66^Zn_plasma-diet_ (and henceforth also including serum) are thus unlikely to result from this. Values from our study roughly match those from Mahan and colleagues’ study, whereby plasma shows similar or slightly lower *δ*^66^Zn values relative to the diet, while RBC exhibits somewhat higher values.^27^ Plasma values from Moynier *et al*. (2013) show large heterogeneity, but the mean *δ*^66^Zn value is also lower relative to the diet.^26^ At the moment, it is unclear why values in sheep from Balter *et al*. (2010) deviate so strongly from the aforementioned trend, whereby RBC's values are much lower than the diet and plasma is much higher.^24^ Not only is this the reverse trend to that reported by other studies, but the relative difference with the diet's value is also much more pronounced. Equally, RBC values from Moynier *et al*. (2013), although showing the same trend of higher values relative to the diet, display a much higher Δ^66^Zn_RBC-diet_ value.^26^ However, it is encouraging that results from our and Mahan's studies show somewhat similar *δ*^66^Zn values for RBC and plasma, especially since Zn is mostly bonded to albumin in both and should therefore be expected to have a similar isotopic composition.^73,74^

The most similar bone-to-diet fractionation is observed in sea breams (see McCormack *et al*., 2024) where the reported offsets between muscle and diet *δ*^66^Zn values (Δ^66^Zn_muscle-diet_ = −0.45 ± 0.03 ‰, (1 σ), *n* = 5) are also similar to the current study (Δ^66^Zn_muscle-diet_ = −0.57 ± 0.10 ‰ (1 σ), *n* = 2).^70^ While the *δ*^66^Zn values of muscle from the current study show the same trend toward lower values relative to the diet as reported for other rodents,^25,26^ the Δ^66^Zn_muscle-diet_ value of rat individuals fed only the supplier's diet (i.e. assumed to be at equilibrium with their diets) is −0.57 ‰, while studies from Balter *et al*. (2013) and Moynier *et al*. (2013) are at −0.21 and −0.18 ‰, respectively.^25,26^ Different developmental stages (and consequently isotopic equilibrium of muscle with the diet) are not assumed to have played a role in differences between studies performed on rodents in *δ*^66^Zn values of muscles. Mice specimens from Balter *et al*. (2013) should be at isotopic equilibrium with their diet, so should the ones from Moynier *et al*. (2013) terminated in a later stage of the staggered-killing sequence.^25,26^ Nonetheless, it is worth mentioning that all controlled feeding experiments conducted so far were different in design, conditions, number of specimens, and with various species. The many differences between them thus could likely explain the variability observed. Moreover, and perhaps more importantly, the relationship between diet and consumers’ *δ*^66^Zn values was not expressively the main objective in other studies. Similarly, the current study was also not designed solely around the isotopic composition of the diet, nor solely for Zn either, but for other isotopic trophic level proxies or diet related dental wear.^48–51^ Some caveats and limitations thus also apply to the current study, as more analyzed specimens for each diet group and a longer experiment duration (primarily to avoid confounding effects from growth performance) could alleviate some uncertainties and strengthen the results.

**Fig. 8:**
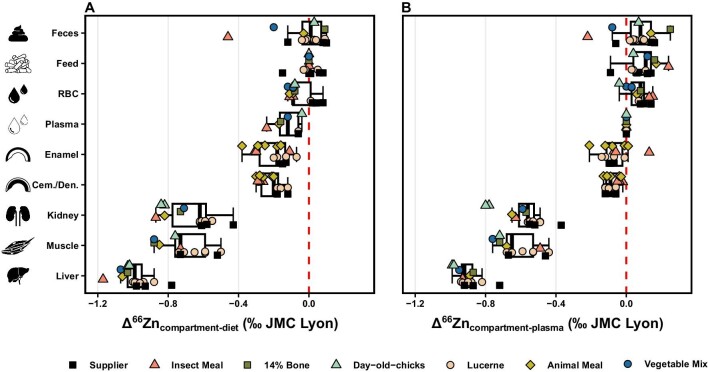
The Zn isotope compositions (‰, relative to the JMC-Lyon Zn isotope standard) of the liver, muscle, kidney, enamel, cementum/dentine (Cem./Den.), plasma, red blood cells (RBC), feed, and feces from the current study relative to a consumer's (A) diet, and (B) plasma (Δ^66^Zn values). Bone and hair are excluded because of their slower equilibration rate. The red dashed line corresponds to the mean feeds’, plasma's, and RBC's values normalized to 0 ‰. Each point shapes and colors are associated with a distinct diet: pelleted supplier's diet, pelleted insect meal diet, pelleted animal meal diet with a 14% bone-meal supplement, day-old-chick diet, pelleted lucerne diet, pelleted animal meal diet, and vegetable mix diet. The boxes represent the 25th–75th percentiles, with the median represented by a bold horizontal line.

To our knowledge, no *δ*^66^Zn values of mammal teeth's bioapatite tissues have been previously analyzed in controlled feeding settings (Figs. 2 and 8), only for sea breams fish.^70^ However, as enamel(oid) is the preferred tissue for paleodietary studies, its isotopic fractionation relative to diet is highly relevant for archeological and paleontological reconstructions of past diets because of this tissue's high degree of mineralization, large bioapatite crystallite size, and low porosity, and hence high resistance to diagenetic alteration.^75,76^ Overall, Δ^66^Zn_enamel-diet_ of rats is −0.18 ‰ ± 0.01 ‰ (1σ, *n* = 17) across diets from Experiment-1 (i.e. pelleted diets), both animal- and plant-based ones, which is similar, albeit slightly lower, to that of sea breams’ enameloid (Δ^66^Zn_enameloid-diet_ = −0.29 ‰ ± 0.03 ‰ (1σ), *n* = 7). As reported elsewhere, the mandibular incisors used in the current study are expected to have been completely replaced over the course of the experiment based on their mean total tooth length and growth rate.^51,77^ Thus, their *δ*^66^Zn values should solely reflect a period when the animal ingested the experimental diet (54 days, after a 5-day acclimatization period during which the animals also received the supplier's feed in addition to the experimental food). The Δ^66^Zn_enamel-bone_ of rat individuals fed only the supplier's diet (i.e. those whose bones are assumed to be at equilibrium with their diets, as their last dietary switch occurred at weaning from breastmilk) is −0.15 ‰ (± 0.04 ‰ (1σ), *n* = 2), which is broadly similar, albeit slightly lower, than previously reported mean Δ^66^Zn_enamel-bone_: −0.2 ‰ in terrestrial mammals from Koobi Fora, −0.18 ‰ in humans, enamel-dentine offset of −0.22 ‰ in fossil terrestrial mammals, and enameloid-osteodentine offset in diverse elasmobranch species of −0.21 ‰.^36,39,42,78^ However, the amplitude of the Δ^66^Zn_enamel-cementum/dentine_ from the current study is lower, −0.10 ‰ (0.08 (1σ), *n* = 14). This is likely the result of cementum, the incisors’ outer hardened layer, being the predominant Zn contribution tissue in those samples, and would suggest similar *δ*^66^Zn values in cementum and enamel.^52^ Nevertheless, the similar Δ^66^Zn_enamel-bone_ value across studies and similar Δ^66^Zn_enamel-diet_ value across diet groups from this study also further support an apparent diet-enamel isotope equilibrium.

Lastly, no tissues, biofluids, or excreta show significant enrichment in the heavy ^66^Zn isotope relative to the diet; when at or close to equilibrium with the diet, all *δ*^66^Zn values are either lower or roughly equal. This differs from other controlled feeding experiments and is somewhat surprising as it suggests a ^66^Zn deficit in the specimens’ Zn balance.^24–26^ While a substantial variety of different tissues, biofluids, or excreta were analyzed, and most Zn is passively held in bone and muscle, we cannot exclude the possibility of other tissues being enriched in ^66^Zn compared with the diet. While urine was not prepared and analyzed in the current study, all our models, along with the various sweeps of the space of parameters (Supplementary Material 2), suggest a potential preferential loss of heavy Zn isotopes in urine, as reported elsewhere.^66^ However, the small Zn efflux in urine (Fig. 3) hardly seems enough to explain the lack of heavy Zn isotope balance. Nonetheless, all *δ*^66^Zn values from hard and soft tissues of the feeding experiment rats are either lower or roughly equal to the diet, thus aligning well with the observed generally lower *δ*^66^Zn values higher up in natural food chains.

### The *δ*^66^Zn values in a rat body and its relation to diet

The pelleted lucerne diet exhibits the lowest *δ*^66^Zn values (i.e. the most similar to a ‘carnivore-like’ diet in a natural food web). It thus differs from the typical Zn trophic level successions from a food web (i.e. *δ*^66^Zn_carnivore_ < *δ*^66^Zn_bone-eating carnivore_ & *δ*^66^Zn_omnivore_ < *δ*^66^Zn_herbivore_), but this is likely due to ingredients of the different diets not being taken from a single context that would mimic real trophic interactions. Nonetheless, the data support trophic discrimination observed in food webs, as muscles and other soft tissues (i.e. those that would be predominantly eaten by carnivores) in rats show low *δ*^66^Zn values relative to their diet, just as carnivores exhibit lower *δ*^66^Zn values than sympatric herbivores.^36,39,40^ Based on the data of specimens fed only the supplier's diet, the muscle-diet spacing is −0.63 ‰, which, when assuming relatively constant tissue fractionation factors between species and muscle as the main digested tissue by carnivorous consumers, would effectively suggest such a trophic spacing for larger mammals between the same tissues of predators and their prey. This notably corresponds well to observed trophic spacing observed for natural food webs in Laos (∼ −0.60 ‰) but is bigger than in others food webs (ranging from ∼ −0.45 ‰ to ∼ −0.32 ‰).^36,37,39–41^ As stated already (Fig. 7), this spacing differs (up to > 3 times) from that of almost all other controlled feeding experiments (Δ^66^Zn_muscle-diet_ = 0.18 ± 0.14 ‰ (1σ), *n* = 4 (Balter *et al.*, 2010); Δ^66^Zn_muscle-diet_ = −0.14 ± 0.06 ‰ (1σ), *n* = 4 (Balter *et al*., 2013); and Δ^66^Zn_muscle-diet_ = −0.10 ± 0.02 ‰ (1σ), *n* = 2 (Moynier *et al*., 2013)), except for McCormack *et al*., 2024 (Δ^66^Zn_muscle-diet_ = −0.45 ± 0.03 ‰, (1 σ), *n* = 5), which is also similar to observed trophic spacing observed for natural food webs.^24–26,70^ However, it is worth noting that those spacings of other controlled feeding experiments differ from every trophic ecology study in natural food webs.^36,37,39–42^

The absence of significant differences in isotopic fractionation during intestinal Zn absorption across diets is seemingly confirmed through the box models as they predict similar *δ*^66^Zn values in the various compartments as those empirically obtained in this study. However, some variability between diets can be observed for most tissues, but can also likely be accounted for by growth performance, as highlighted in Fig. 4, and by counting statistics, as the number of individuals between groups varied in the current study. Specifically, the pelleted lucerne and pelleted supplier's diets have a higher count of individuals analyzed in the current study and exhibit similar Δ^66^Zn_compartment-diet_ values (Table S5). Simple simulations (Figure S2) using randomly selected pairs of *δ*^66^Zn values of feed (*n* = 3) and muscle tissues (*n* = 6) from the pelleted lucerne diet show that considerable Δ^66^Zn_muscle-diet_ value differences (from −0.79 to −0.48 ‰) can be obtained when using a single specimen. When also accounting for variability induced by different growth performances, it becomes evident that variability across diets can ensue. Moreover, the *δ*^66^Zn values of each tissue (except for bone) are also directly dependent on the composition of their associated diet (Figure S3) and follow a roughly 1:1 slope, further supporting similar isotopic fractionation across diets.

The differences between the pelleted lucerne diet with the other pelleted diets could be tentatively associated with previous assumptions that preferential precipitation of light Zn isotopes with phytates in the intestinal tract induces higher *δ*^66^Zn in plant-matter consumers than in animal-matter ones.^36^ However, phytate content, estimated for three pelleted diets of the Basic Experiment, is low (0.06, 0.10, and 0.11%, respectively for the pelleted lucerne, animal meal, and insect meal diets) and comparable only to amounts in the very low range found in food items.^79^ Phytate content thus can be ruled out as a source of Δ^66^Zn_compartment-diet_ variability between diets. While differences between diets from Experiment-1 could be more generally associated with plant- vs. animal-based diets since animal proteins are seemingly associated with a higher Zn uptake and improved Zn bioavailability, this is not supported by Δ^66^Zn_compartment-diet_ values of various tissues and biofluids from individuals of the Natural Diets experiment (Experiment 3).^18,20,21^ Moreover, the natural diets not only have more homogeneous proportions of secondary ingredients (i.e. all ingredients other than the ‘defining’ one such as lucerne, animal meal, etc.) compared with those of Experiment-1 and 2 but the day-old-chicks diet is also almost solely composed of animal matter (whole frozen day-old chicks and supplement; Table S4) as opposed to the pelleted animal meal (25%), insect meal (26%), and 14% Bone (35.5%, which includes the lamb and bone meal) diets, respectively (Table S3). Consequently, they offer a much better comparison of the impact of animal- and plant-matter in the diet relative to isotopic fractionation upon intestinal absorption. While this experiment was much shorter than Experiment-1, the faster-turning tissues (e.g. plasma, RBC, kidney, and liver) nonetheless offer the chance to compare ∆^66^Zn_compartment-diet_ values at or roughly at equilibrium with the diet (Fig. 8 and Table S5). The differences here are small, and the highest values are not systematically associated with the same diet.

The isotopic composition of the dietary Zn intake as primary control over the *δ*^66^Zn values of animal tissues is also illustrated in the Bone Addition Experiment, for which the value of the pelleted animal meal (*δ*^66^Zn = −0.09 ‰) was shifted toward that of the bone-meal supplement (*δ*^66^Zn = 0.96 ‰); this resulted in higher *δ*^66^Zn values in the pelleted bone addition meal (*δ*^66^Zn = 0.00 ‰) and, accordingly, also in the tissues and biofluids of its consumer (Figure S1). This observed difference supports data reported in other studies, where bone-eating carnivores’ *δ*^66^Zn values are distinct from sympatric carnivores, and omnivores have intermediate *δ*^66^Zn values between carnivores and herbivores.^36,39,40,44,80^ Both cases suggest that the mixing of all resources eaten (in one case, soft and hard animal tissues, and in the other animal and plant-matter) dictates *δ*^66^Zn values in consumers, without any strong bias for given food items (e.g. animal or plant-matter). The Bone Addition Experiment supports this assumption, whereby a 14% addition of bone-meal supplement contributed to roughly 9% of the *δ*^66^Zn value of the feed, with both supplement and original feed having a similar Zn concentration of 78 and 67 μg/g, respectively (Table S1). This was already suspected because of the isotopically distinct *δ*^66^Zn range of values recorded for omnivore species in Late Pleistocene fossil mammal assemblages of Laos, which suggested that the averaged Zn isotope composition of the diet was the primary reason for recorded values in a consumer.^39,40^ While the average isotopic composition of the dietary Zn intake will undoubtedly still depend on factors such as Zn concentration and Zn bioavailability of the different ingested food items, the absence of any marked differences suggests minimal isotopic fractionation upon Zn bio-assimilation between animal- (soft and hard tissues) and plant-matter or of significant bias toward either of those resource types. This is especially promising for (paleo)dietary reconstructions as it suggests that *δ*^66^Zn values recorded in tissues could be used to trace the averaged Zn isotope composition of the diet or simply add nuance to dietary interpretation, relying less on stark differences (e.g. trophic level differences and type/degree of protein consumption) and more on components in the diet themselves.

**Fig. 9:**
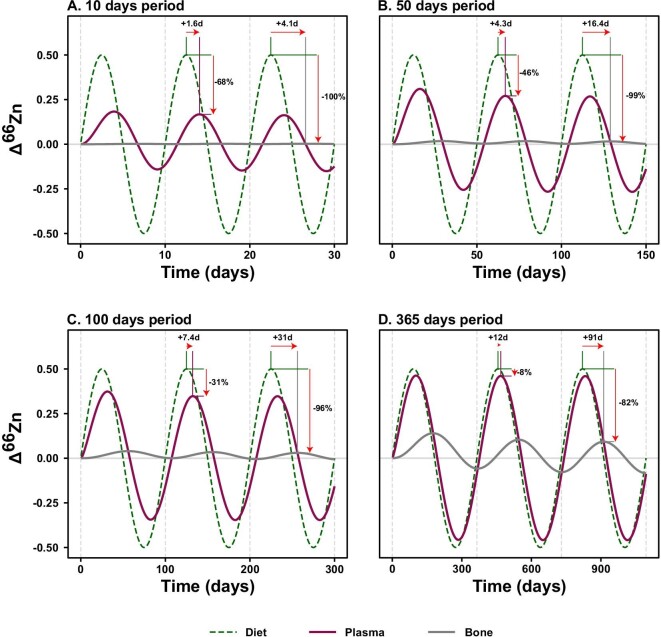
Transfer of sinusoidal variations of dietary δ^66^Zn to rat plasma and bone for 10, 50, 100, and 365 days periods. The shifts in phase (in days) are shown at local maxima for both plasma and bone relative to the diet, and the buffering of the signal relative to diet as the proportion of lost total amplitude (in %). Isotopic compositions are shown as ∆^66^Zn deviation from initial steady-state compositions in ‰ (relative to the JMC-Lyon Zn isotope standard).

Finally, we show the expected transfer of a sinusoidal (e.g. seasonal) variation of diet *δ*^66^Zn to a rapid reservoir (plasma) and a slow reservoir (bone) based on the Zn cycle of an adult rat, as assessed in this study (Fig. 9). The dynamic response of the organism to various periodical shifts in diet isotope compositions, ranging from 10 days to a year, shows distinct sensitivity of plasma (more sensitive) and bone (less sensitive) to changes in dietary sources.

For example, for short periods of 10 days, the Zn isotope variations in plasma are buffered by ca. 68% and shifted in time by about 2 days (Fig. 9A), against 8% and 12 days for yearly variations of diet *δ*^66^Zn (Fig. 9D). When taking into account durations required to reach specific isotopic equilibration thresholds (Fig. 4 and Table 4), these simulations bring new constraints to the possibility of tracking intra-individual dietary changes, especially in incrementally growing tissues like enamel, which is the geochemical archive of choice in paleobiology. Although the prediction of isotopic variations in enamel requires the modeling of additional processes affecting the signal (e.g. enamel secretion and maturation, tooth geometry, or sampling resolution, etc.), such results illustrate the typical responses (i.e. phase shift and buffering) of plasma, which is the primary source of Zn during enamel formation.^61^ Comparatively, the whole bone Zn reservoir displays a much lower sensitivity to short-term variations in isotopic compositions of dietary sources. These modeled transfers of sinusoidal variations of dietary *δ*^66^Zn to rat plasma and bone thus efficiently illustrate additional considerations that need to be accounted for in animals experiencing shifts in diet isotope compositions (for example, either from season-based availability of different resources in a natural food web or from dietary switch in a controlled feeding experiment).

## Conclusion

In the current study, substantial and systematic fractionation of Zn isotopes was reported across tissues, biofluids, and excreta of rats fed during controlled feeding experiments with diets containing different types and amounts of plant- and animal-matter. The evolution of *δ*^66^Zn values in both soft and hard tissues was explored using a box-model approach, notably allowing for assessing the time required to fully equilibrate the main Zn reservoirs of the organism with dietary Zn. In turn, this demonstrated that most tissues and biofluids of the rats were almost fully equilibrated (∼90%) after about 2 months, except for bone and hair, whose Zn residence time was much longer than expected from literature data. The different diets induce similar Δ^66^Zn_compartment-diet_ values, whereby the *δ*^66^Zn values seem to primarily reflect the dietary Zn intake, although growth performances appear to induce some variability. In particular, the Δ^66^Zn_muscle-diet_ value from the current study is lower than in other experimental studies but more consistent with the trophic spacing between predators and their prey observed in natural food webs. Contrary to expectation, no marked distinction between animal- and plant-based diets could be seen, suggesting a similar Zn isotope fractionation upon intestinal absorption. This is consequently of great interest for (paleo)dietary reconstructions as it suggests a fairly unbiased average in the isotopic composition of the dietary Zn intake of a consumer and its tissues, likely allowing for more refined dietary interpretations. Lastly, the similar Zn isotope fractionation between different diets and enamel analyzed from controlled feeding experiments is equally of great importance for (paleo)dietary studies, as it paves the way for actual dietary reconstruction beyond relative trophic positions between individuals or dietary groups using diet-related *δ*^66^Zn values of taphonomically robust enamel from fossil teeth.

## Supplementary Material

mfae026_Supplemental_File

## Data Availability

The data underlying this article are available in the article and in its online supplementary material.
