# Supplementary material for: Dietary and homeostatic controls of Zn isotopes in rats: a controlled feeding experiment and modeling approach
Source: Metallomics. 2024 May 16;16(6):mfae026. doi: 10.1093/mtomcs/mfae026 (PMC11157155; doi:10.1093/mtomcs/mfae026)
Supplement: mfae026_Supplemental_File [file mfae026_supplemental_file.docx]

**Supplementary Material to**

**Dietary and homeostatic controls of Zn isotopes in rats: A controlled-feeding experiment study**

Nicolas Bourgon, Théo Tacail, Klervia Jaouen, Jennifer N. Leichliter, Jeremy McCormack, Daniela E. Winkler, Marcus Clauss, Thomas Tütken

This file contains:

Supplementary Material-1: Tables and Figures

Supplementary Material-2: Sweep CI fits

Supplementary Material-3: Dietary switch figures

# ****Supplementary Material-1: Tables and Figures****

| **S-EVA #** | **Individual** | **Diet** | **Sample type** | ***δ*^66^Zn (‰)** | ***δ*^67^Zn (‰)** | ***δ*^68^Zn (‰)** | **[Zn] (µg/g)** | **Zn cont. (µg)** |
| --- | --- | --- | --- | --- | --- | --- | --- | --- |
| 37603 | 1ARA4B1 | Insect Meal | Cementum/Dentine | -0.3 | -0.29 | -0.43 | 120 | 793 |
| 35761 | 1ARA4B1 | Insect Meal | Enamel | -0.15 | 0.11 | -0.1 | 127 | 610 |
| 35106 | 1ARA4B2 | Insect Meal | Bone | 0.26 | 0.3 | 0.55 | 101 | 3332 |
| 37604 | 1ARA4B2 | Insect Meal | Cementum/Dentine | -0.32 | -0.36 | -0.49 | 120 | 729 |
| 37623 | 1ARA4B2 | Insect Meal | Enamel | -0.35 |  |  | 58 | 292 |
| 35225 | 1ARA4B2 | Insect Meal | Feces | -0.5 | -0.77 | -0.96 | 229 | 11508 |
| 35229 | 1ARA4B2 | Insect Meal | Hair | -0.02 | 0.01 | -0.03 | 163 | 8135 |
| 35233 | 1ARA4B2 | Insect Meal | Kidney | -0.91 | -1.34 | -1.63 | 100 | 5047 |
| 35237 | 1ARA4B2 | Insect Meal | Liver | -1.21 | -1.75 | -2.3 | 92 | 4631 |
| 35241 | 1ARA4B2 | Insect Meal | Muscle | -0.77 | -1.1 | -1.48 | 33 | 1663 |
| 35253 | 1ARA4B2 | Insect Meal | Plasma | -0.28 | -0.05 | -0.32 | 1 | 523 |
| 35259 | 1ARA4B2 | Insect Meal | Red Blood Cells | -0.13 | -0.18 | -0.25 | 3 | 3485 |
| 35338 | 1ARA4B2 | Insect Meal | Red Blood Cells | -0.15 | -0.07 | -0.22 | 0 | 175 |
| 37608 | 1ARA4G1 | Insect Meal | Cementum/Dentine | -0.33 | -0.32 | -0.48 | 118 | 611 |
| 37624 | 1ARA4G1 | Insect Meal | Enamel | -0.34 |  |  | 69 | 282 |
| 35107 | 1ARA5B1 | Animal Meal | Bone | 0.22 | 0.37 | 0.48 | 81 | 2625 |
| 35226 | 1ARA5B1 | Animal Meal | Feces | -0.12 | -0.16 | -0.22 | 265 | 13276 |
| 35230 | 1ARA5B1 | Animal Meal | Hair | 0.1 | 0.17 | 0.18 | 161 | 8040 |
| 35234 | 1ARA5B1 | Animal Meal | Kidney | -0.91 | -1.33 | -1.54 | 86 | 4361 |
| 35238 | 1ARA5B1 | Animal Meal | Liver | -1.15 | -1.64 | -2.19 | 90 | 4572 |
| 35242 | 1ARA5B1 | Animal Meal | Muscle | -0.94 | -1.29 | -1.77 | 30 | 1510 |
| 35254 | 1ARA5B1 | Animal Meal | Plasma | -0.26 | -0.21 | -0.4 | 1 | 745 |
| 35258 | 1ARA5B1 | Animal Meal | Red Blood Cells | -0.18 | -0.24 | -0.32 | 6 | 5409 |
| 35337 | 1ARA5B1 | Animal Meal | Red Blood Cells | -0.2 | -0.13 | -0.36 | 0 | 307 |
| 37605 | 1ARA5B2 | Animal Meal | Cementum/Dentine | -0.39 | -0.35 | -0.59 | 152 | 773 |
| 35763 | 1ARA5B2 | Animal Meal | Enamel | -0.27 | -0.19 | -0.33 | 142 | 680 |
| 37609 | 1ARA5G1 | Animal Meal | Cementum/Dentine | -0.35 | -0.42 | -0.61 | 125 | 737 |
| 35765 | 1ARA5G1 | Animal Meal | Enamel | -0.25 | -0.2 | -0.32 | 127 | 751 |
| 37610 | 1ARA5G2 | Animal Meal | Cementum/Dentine | -0.37 | -0.45 | -0.62 | 123 | 700 |
| 37625 | 1ARA5G2 | Animal Meal | Enamel | -0.47 |  |  | 89 | 514 |
| 37613 | 1ARA5R1 | Animal Meal | Cementum/Dentine | -0.3 | -0.24 | -0.41 | 110 | 583 |
| 37626 | 1ARA5R1 | Animal Meal | Enamel | -0.34 |  |  | 76 | 350 |
| 37614 | 1ARA5R2 | Animal Meal | Cementum/Dentine | -0.29 | -0.22 | -0.49 | 137 | 806 |
| 37627 | 1ARA5R2 | Animal Meal | Enamel | -0.38 |  |  | 82 | 495 |
| 35105 | 1ARB1B1 | Lucerne | Bone | 0.23 | 0.52 | 0.48 | 125 | 2518 |
| 37606 | 1ARB1B1 | Lucerne | Cementum/Dentine | -0.42 | -0.51 | -0.66 | 131 | 692 |
| 37620 | 1ARB1B1 | Lucerne | Enamel | -0.43 |  |  | 141 | 663 |
| 35119 | 1ARB1B1 | Lucerne | Feces | -0.27 | -0.36 | -0.47 | 143 | 4346 |
| 35249 | 1ARB1B1 | Lucerne | Hair | -0.01 | 0.02 | 0.01 | 185 | 5055 |
| 35113 | 1ARB1B1 | Lucerne | Kidney | -0.92 | -1.31 | -1.74 | 36 | 3111 |
| 35110 | 1ARB1B1 | Lucerne | Liver | -1.3 | -1.88 | -2.53 | 53 | 4965 |
| 35116 | 1ARB1B1 | Lucerne | Muscle | -1.04 | -1.6 | -1.97 | 21 | 1568 |
| 35133 | 1ARB1B1 | Lucerne | Plasma | -0.36 | -0.19 | -0.59 |  |  |
| 35132 | 1ARB1B1 | Lucerne | Red Blood Cells | -0.29 | -0.51 | -0.56 |  |  |
| 36027 | 1ARB1B2 | Lucerne | Bone | 0.23 | 0.47 | 0.53 | 153 | 1625 |
| 37602 | 1ARB1B2 | Lucerne | Cementum/Dentine | -0.47 | -0.61 | -0.81 | 238 | 1263 |
| 37619 | 1ARB1B2 | Lucerne | Enamel | -0.37 |  |  | 1497 | 5690 |
| 36034 | 1ARB1B2 | Lucerne | Feces | -0.31 | -0.39 | -0.51 | 201 | 1119 |
| 36044 | 1ARB1B2 | Lucerne | Hair | 0.13 | 0.34 | 0.36 | 181 | 1083 |
| 36737 | 1ARB1B2 | Lucerne | Kidney | -0.85 | -1.11 | -1.45 | 82 | 1234 |
| 36744 | 1ARB1B2 | Lucerne | Liver | -1.18 | -1.7 | -2.21 | 89 | 1329 |
| 36750 | 1ARB1B2 | Lucerne | Muscle | -0.89 | -1.2 | -1.55 | 31 | 785 |
| 36028 | 1ARB1G1 | Lucerne | Bone | 0.28 | 0.47 | 0.61 | 155 | 2637 |
| 37607 | 1ARB1G1 | Lucerne | Cementum/Dentine | -0.48 | -0.47 | -0.76 | 123 | 639 |
| 37621 | 1ARB1G1 | Lucerne | Enamel | -0.48 |  |  | 115 | 577 |
| 36035 | 1ARB1G1 | Lucerne | Feces | -0.21 | -0.22 | -0.33 | 193 | 970 |
| 36045 | 1ARB1G1 | Lucerne | Hair | 0.1 | 0.32 | 0.37 | 188 | 1198 |
| 36738 | 1ARB1G1 | Lucerne | Kidney | -0.92 | -1.28 | -1.53 | 64 | 954 |
| 36745 | 1ARB1G1 | Lucerne | Liver | -1.28 | -1.81 | -2.39 | 85 | 1275 |
| 36751 | 1ARB1G1 | Lucerne | Muscle | -1.01 | -1.44 | -1.76 | 25 | 614 |
| 36029 | 1ARB1G2 | Lucerne | Bone | 0.3 | 0.44 | 0.65 | 158 | 1678 |
| 37611 | 1ARB1G2 | Lucerne | Cementum/Dentine | -0.48 | -0.5 | -0.81 | 110 | 629 |
| 37622 | 1ARB1G2 | Lucerne | Enamel | -0.5 |  |  | 94 | 556 |
| 36036 | 1ARB1G2 | Lucerne | Feces | -0.32 | -0.4 | -0.54 | 234 | 1219 |
| 36046 | 1ARB1G2 | Lucerne | Hair | 0.08 | 0.27 | 0.32 | 187 | 1186 |
| 36739 | 1ARB1G2 | Lucerne | Kidney | -0.91 | -1.33 | -1.62 | 76 | 1145 |
| 36746 | 1ARB1G2 | Lucerne | Liver | -1.24 | -1.75 | -2.33 | 84 | 1264 |
| 36752 | 1ARB1G2 | Lucerne | Muscle | -0.89 | -1.2 | -1.56 | 33 | 837 |
| 36030 | 1ARB1R1 | Lucerne | Bone | 0.25 | 0.43 | 0.56 | 158 | 1675 |
| 36042 | 1ARB1R1 | Lucerne | Feces | -0.34 | -0.47 | -0.57 | 197 | 1217 |
| 36047 | 1ARB1R1 | Lucerne | Hair | 0.13 | 0.35 | 0.39 | 183 | 1188 |
| 36740 | 1ARB1R1 | Lucerne | Kidney | -0.85 | -1.2 | -1.55 | 80 | 1195 |
| 36747 | 1ARB1R1 | Lucerne | Liver | -1.29 | -1.82 | -2.44 | 92 | 1384 |
| 36753 | 1ARB1R1 | Lucerne | Muscle | -0.95 | -1.32 | -1.65 | 27 | 679 |
| 36031 | 1ARB1R2 | Lucerne | Bone | 0.2 | 0.34 | 0.47 | 159 | 2227 |
| 37612 | 1ARB1R2 | Lucerne | Cementum/Dentine | -0.46 | -0.52 | -0.75 | 128 | 716 |
| 36043 | 1ARB1R2 | Lucerne | Feces | -0.26 | -0.28 | -0.42 | 280 | 1368 |
| 36048 | 1ARB1R2 | Lucerne | Hair | 0.17 | 0.42 | 0.5 | 157 | 813 |
| 36741 | 1ARB1R2 | Lucerne | Kidney | -0.89 | -1.27 | -1.46 | 59 | 886 |
| 36748 | 1ARB1R2 | Lucerne | Liver | -1.26 | -1.8 | -2.37 | 85 | 1281 |
| 36754 | 1ARB1R2 | Lucerne | Muscle | -0.8 | -1.08 | -1.36 | 31 | 768 |
| 36026 | 1BRA1B1 | Supplier | Bone | 0.46 | 0.74 | 0.96 | 139 | 1416 |
| 37616 | 1BRA1B1 | Supplier | Cementum/Dentine | 0.24 |  |  | 117 | 584 |
| 37615 | 1BRA1B1 | Supplier | Enamel | 0.29 |  |  | 80 | 488 |
| 36033 | 1BRA1B1 | Supplier | Feces | 0.52 | 0.93 | 1.14 | 121 | 758 |
| 36736 | 1BRA1B1 | Supplier | Kidney | -0.01 | 0.06 | 0.15 | 63 | 948 |
| 36743 | 1BRA1B1 | Supplier | Liver | -0.36 | -0.46 | -0.57 | 64 | 956 |
| 36749 | 1BRA1B1 | Supplier | Muscle | -0.1 | -0.01 | 0.15 | 31 | 781 |
| 36025 | 1BRC1B1 | Supplier | Bone | 0.39 | 0.64 | 0.83 | 135 | 1409 |
| 37618 | 1BRC1B1 | Supplier | Cementum/Dentine | 0.3 |  |  | 158 | 837 |
| 37617 | 1BRC1B1 | Supplier | Enamel | 0.27 |  |  | 157 | 847 |
| 36032 | 1BRC1B1 | Supplier | Feces | 0.3 | 0.52 | 0.65 | 208 | 1137 |
| 36735 | 1BRC1B1 | Supplier | Kidney | -0.16 | -0.13 | -0.14 | 68 | 1021 |
| 36742 | 1BRC1B1 | Supplier | Liver | -0.51 | -0.7 | -0.82 | 73 | 1097 |
| 35223 | 1BRC4B1 | Supplier | Bone | 0.27 | 0.47 | 0.52 | 140 | 2818 |
| 35227 | 1BRC4B1 | Supplier | Feces | 0.5 | 0.77 | 0.97 | 151 | 7490 |
| 35231 | 1BRC4B1 | Supplier | Hair | 0.08 | 0.12 | 0.2 | 208 | 9616 |
| 35235 | 1BRC4B1 | Supplier | Kidney | -0.19 | -0.2 | -0.28 | 97 | 4921 |
| 35239 | 1BRC4B1 | Supplier | Liver | -0.56 | -0.78 | -1.08 | 87 | 4414 |
| 35243 | 1BRC4B1 | Supplier | Muscle | -0.31 | -0.42 | -0.54 | 64 | 3224 |
| 35255 | 1BRC4B1 | Supplier | Plasma | 0.36 | 0.96 | 0.93 | 1 | 305 |
| 35257 | 1BRC4B1 | Supplier | Red Blood Cells | 0.46 | 0.69 | 0.92 | 4 | 3161 |
| 35336 | 1BRC4B1 | Supplier | Red Blood Cells | 0.44 | 0.8 | 0.88 | 0 | 133 |
| 35340 | 1BRC4B1 | Supplier | Red Blood Cells | 0.5 | 0.74 | 1.02 | 5 | 1351 |
| 35108 | 1ERB6B2 | 14% Bone | Bone | 0.32 | 0.57 | 0.69 | 73 | 2183 |
| 35120 | 1ERB6B2 | 14% Bone | Feces | 0.09 | 0.12 | 0.23 | 157 | 4730 |
| 35131 | 1ERB6B2 | 14% Bone | Hair | 0.07 | -0.01 | 0.16 | 85 | 9105 |
| 35114 | 1ERB6B2 | 14% Bone | Kidney | -0.73 | -1.09 | -1.37 | 60 | 5256 |
| 35111 | 1ERB6B2 | 14% Bone | Liver | -1.03 | -1.6 | -1.99 | 38 | 3555 |
| 35117 | 1ERB6B2 | 14% Bone | Muscle | -0.88 | -1.27 | -1.66 | 23 | 1826 |
| 35136 | 1ERB6B2 | 14% Bone | Plasma | -0.16 | 0.41 | -0.14 | 2 |  |
| 35135 | 1ERB6B2 | 14% Bone | Red Blood Cells | -0.08 | -0.15 | -0.11 | 8 |  |
| 35224 | 2AA1G1 | Vegetable Mix | Bone | 0.37 | 0.58 | 0.72 | 139 | 2820 |
| 35228 | 2AA1G1 | Vegetable Mix | Feces | 0.25 | 0.45 | 0.55 | 129 | 6530 |
| 35232 | 2AA1G1 | Vegetable Mix | Hair | 0.23 | 0.32 | 0.46 | 191 | 9563 |
| 35250 | 2AA1G1 | Vegetable Mix | Hair | 0.18 | 0.27 | 0.37 | 164 | 8254 |
| 35236 | 2AA1G1 | Vegetable Mix | Kidney | -0.26 | -0.36 | -0.47 | 91 | 4591 |
| 35240 | 2AA1G1 | Vegetable Mix | Liver | -0.62 | -0.9 | -1.2 | 91 | 4619 |
| 35244 | 2AA1G1 | Vegetable Mix | Muscle | -0.43 | -0.49 | -0.68 | 38 | 1923 |
| 35256 | 2AA1G1 | Vegetable Mix | Plasma | 0.33 | 0.76 | 0.71 | 1 | 764 |
| 35260 | 2AA1G1 | Vegetable Mix | Red Blood Cells | 0.36 | 0.56 | 0.71 | 7 | 6032 |
| 35339 | 2AA1G1 | Vegetable Mix | Red Blood Cells | 0.33 | 0.7 | 0.69 | 0 | 126 |
| 35341 | 2AA1G1 | Vegetable Mix | Red Blood Cells | 0.36 | 0.54 | 0.73 | 6 | 1543 |
| 35109 | 2AA6B1 | Day-old-chicks | Bone | 0.33 | 0.54 | 0.71 | 115 | 2692 |
| 35141 | 2AA6B1 | Day-old-chicks | Bone | 0.28 | 0.46 | 0.58 | 92 |  |
| 35121 | 2AA6B1 | Day-old-chicks | Feces | 0.34 | 0.42 | 0.73 | 312 | 9972 |
| 35122 | 2AA6B1 | Day-old-chicks | Hair | 0.19 | 0.13 | 0.42 | 78 | 7815 |
| 35115 | 2AA6B1 | Day-old-chicks | Kidney | -0.51 | -0.72 | -0.97 | 55 | 4642 |
| 35143 | 2AA6B1 | Day-old-chicks | Kidney | -0.53 | -0.82 | -1.03 | 75 |  |
| 35112 | 2AA6B1 | Day-old-chicks | Liver | -0.72 | -1.1 | -1.37 | 121 | 4139 |
| 35142 | 2AA6B1 | Day-old-chicks | Liver | -0.71 | -1.09 | -1.38 | 54 |  |
| 35118 | 2AA6B1 | Day-old-chicks | Muscle | -0.45 | -0.58 | -0.86 | 21 | 1569 |
| 35140 | 2AA6B1 | Day-old-chicks | Plasma | 0.27 | 0.73 | 0.63 | 5 |  |
| 35139 | 2AA6B1 | Day-old-chicks | Red Blood Cells | 0.23 | 0.29 | 0.49 | 9 |  |
| 35129 |  | 14% Bone | Feed | 0 | 0.09 | 0.15 | 88 | 2658 |
| 35127 |  | Animal Meal | Feed | -0.09 | -0.1 | -0.12 | 67 | 2189 |
| 35130 |  | Bone meal | Ingredient | 0.96 | 1.33 | 1.92 | 78 | 3834 |
| 35125 |  | Day-old-chicks | Feed | 0.31 | 0.58 | 0.73 | 32 | 977 |
| 35128 |  | Insect Meal | Feed | -0.04 | -0.07 | -0.01 | 69 | 2144 |
| 35124 |  | Lucerne | Feed | -0.25 | -0.35 | -0.45 | 48 | 1484 |
| 36040 |  | Lucerne | Feed | -0.32 | -0.41 | -0.53 | 106 | 2168 |
| 36041 |  | Lucerne | Feed | -0.33 | -0.42 | -0.53 | 101 | 2044 |
| 35126 |  | Supplier | Feed | 0.27 | 0.44 | 0.53 | 37 | 1219 |
| 35248 |  | Supplier | Feed | 0.43 | 0.65 | 0.9 | 70 | 3493 |
| 36037 |  | Supplier | Feed | 0.5 | 0.81 | 1.11 | 79 | 2029 |
| 36038 |  | Supplier | Feed | 0.48 | 0.78 | 1.14 | 67 | 1354 |
| 36039 |  | Supplier | Feed | 0.42 | 0.84 | 0.99 | 70 | 1484 |
| 35245 |  | Vegetable Mix | Feed | 0.45 | 0.84 | 0.92 | 24 | 1192 |

**Table S1. Full list of samples analyzed for *δ*^66^Zn, with corresponding laboratory internal S-EVA number, original sample number (i.e., rat specimens’ ID), dietary group, and sample type (mostly tissues, excreta, and biofluids, but also the feed and the calcium-rich supplement used in the Bone Addition experiment, listed as "Ingredient"). Abbreviations: S-EVA = Stable isotope-Evolutionary Anthropology (the numbering system used in the Human Evolution department of the Max Planck Institute for Evolution Anthropology, Leipzig, for every sample analyzed for isotopic analysis).**

| **Individual** | **Age (weeks)** | **Diet** | **Initial weight (g)** | **Final weight (g)** | **Max. weight (g)** |
| --- | --- | --- | --- | --- | --- |
| 1ARA4B1 | 20 | Insect Meal | 217 | 266 | 269 |
| 1ARA4B2 | 20 | Insect Meal | 198 | 261 | 261 |
| 1ARA4G1 | 20 | Insect Meal | 204 | 252 | 256 |
| 1ARA5B1 | 20 | Animal Meal | 209 | 274 | 274 |
| 1ARA5B2 | 20 | Animal Meal | 185 | 236 | 236 |
| 1ARA5G1 | 20 | Animal Meal | 193 | 232 | 239 |
| 1ARA5G2 | 20 | Animal Meal | 198 | 251 | 264 |
| 1ARA5R1 | 20 | Animal Meal | 170 | 228 | 229 |
| 1ARA5R2 | 20 | Animal Meal | 205 | 267 | 268 |
| 1ARB1B1 | 20 | Lucerne | 190 | 234 | 246 |
| 1ARB1B2 | 20 | Lucerne | 181 | 211 | 211 |
| 1ARB1G1 | 20 | Lucerne | 205 | 243 | 249 |
| 1ARB1G2 | 20 | Lucerne | 210 | 246 | 249 |
| 1ARB1R1 | 20 | Lucerne | 192 | 225 | 226 |
| 1ARB1R2 | 20 | Lucerne | 192 | 234 | 235 |
| 1BRA1B1 | 12 | Supplier | 195 | 220 | 220 |
| 1BRC1B1 | 12 | Supplier | 218 | 229 | 229 |
| 1BRC4B1 | 12 | Supplier | 193 | 201 | 201 |
| 1ERB6B2 | 20 | 14% Bone | 199 | 233 | 234 |
| 2AA1G1 | 16 | Vegetable Mix | 226 | 251 | 251 |
| 2AA6B1 | 16 | Day-old-chicks | 202 | 230 | 236 |

**Table S2.**  **Full list of weight (in g) for each specimen at the start of the experiments and at termination, with maximal weight as some body mass peaked and then decreased slightly.**

|  | **Proportion (%)** | | | |
| --- | --- | --- | --- | --- |
| **Ingredient** | **Lucerne** | **Animal-meal** | **Insect-meal** | **14% Bone** |
| Main ingredient (lucerne flour (1), lamb meat (2), and protix insect meal (3)) | 56^(1)^ | 25^(2)^ | 26^(3)^ | 21.5^(2)^ |
| Potato protein | 13.5 | 0 | 0 | 0 |
| Ground wheat | 10.23 | 17.68 | 15.95 | 15.21 |
| Ground oat | 7 | 15.5 | 18 | 13.33 |
| Apple pomace | 4.5 | 15 | 14 | 12.9 |
| Beet molasses | 3 | 3 | 3 | 2.58 |
| Soy hulls | 0 | 13 | 10 | 11.18 |
| Straw flour | 0 | 9 | 10 | 7.74 |
| Soy oil | 2.8 | 0 | 0 | 0 |
| Monosodium phosphate | 0.95 | 0 | 0.3 | 0 |
| Carbonate lime | 0 | 0 | 0.8 | 0 |
| Vitamin/Mineral/Amino acid premix | 1.52 | 1.52 | 1.60 | 1.31 |
| Salt | 0.5 | 0.3 | 0.35 | 0.26 |
| Bone meal supplement | 0 | 0 | 0 | 14 |

**Table S3. Full list of ingredients for Experiment 1 (Basic Diet) and 3 (Bone Addition) with their relative proportion (%) of the composition of the whole feed.**

|  | **Vegetable mix** | **Day-old-chicks** |
| --- | --- | --- |
| **Ingredient** | **(%)** | **(%)** |
| Celery Root | 28.97 | 0 |
| Carrot | 19.04 | 0 |
| Stalk celery | 9.82 | 0 |
| Kohlrabi | 9.65 | 0 |
| Parsley | 9.11 | 0 |
| Spinach | 8.11 | 0 |
| Whole frozen day-old chicks | 0 | 100 |
| Quark | 7.3 | 8.3 |
| Sunflower oil | 1.2 | 0 |
| Supplement powder | 0.8 | 3.0 |

**Table S4. Relative proportion (weight %) of the composition of (including supplementations: quark, sunflower oil, and supplement powders) experimental diets for Experiment 3 (natural diets).**

| **S-EVA #** | **Individual** | **Diet** | **Sample type** | **Δ^66^Zn_diet_** | **Δ^66^Zn_plasma_** |
| --- | --- | --- | --- | --- | --- |
| 35108 | 1ERB6B2 | 14% Bone | Bone | 0.32 | 0.48 |
| 35120 | 1ERB6B2 | 14% Bone | Feces | 0.09 | 0.25 |
| 35131 | 1ERB6B2 | 14% Bone | Hair | 0.07 | 0.23 |
| 35114 | 1ERB6B2 | 14% Bone | Kidney | -0.73 | -0.57 |
| 35111 | 1ERB6B2 | 14% Bone | Liver | -1.03 | -0.87 |
| 35117 | 1ERB6B2 | 14% Bone | Muscle | -0.88 | -0.72 |
| 35136 | 1ERB6B2 | 14% Bone | Plasma | -0.16 | 0.00 |
| 35135 | 1ERB6B2 | 14% Bone | RBC | -0.08 | 0.08 |
| 35129 |  | 14% Bone | Feed | 0.00 | 0.16 |
| 35107 | 1ARA5B1 | Animal Meal | Bone | 0.31 | 0.48 |
| 35226 | 1ARA5B1 | Animal Meal | Feces | -0.03 | 0.14 |
| 35230 | 1ARA5B1 | Animal Meal | Hair | 0.19 | 0.36 |
| 35234 | 1ARA5B1 | Animal Meal | Kidney | -0.82 | -0.65 |
| 35238 | 1ARA5B1 | Animal Meal | Liver | -1.06 | -0.89 |
| 35242 | 1ARA5B1 | Animal Meal | Muscle | -0.85 | -0.68 |
| 35254 | 1ARA5B1 | Animal Meal | Plasma | -0.17 | 0.00 |
| 35258 | 1ARA5B1 | Animal Meal | Cem./Den. | -0.09 | 0.08 |
| 35337 | 1ARA5B1 | Animal Meal | Cem./Den. | -0.11 | 0.06 |
| 37605 | 1ARA5B2 | Animal Meal | Cem./Den. | -0.30 | -0.13 |
| 35763 | 1ARA5B2 | Animal Meal | Enamel | -0.18 | -0.01 |
| 37609 | 1ARA5G1 | Animal Meal | Cem./Den. | -0.26 | -0.09 |
| 35765 | 1ARA5G1 | Animal Meal | Enamel | -0.16 | 0.01 |
| 37610 | 1ARA5G2 | Animal Meal | Cem./Den. | -0.28 | -0.11 |
| 37625 | 1ARA5G2 | Animal Meal | Enamel | -0.38 | -0.21 |
| 37613 | 1ARA5R1 | Animal Meal | Cem./Den. | -0.21 | -0.04 |
| 37626 | 1ARA5R1 | Animal Meal | Enamel | -0.25 | -0.08 |
| 37614 | 1ARA5R2 | Animal Meal | Cem./Den. | -0.20 | -0.03 |
| 37627 | 1ARA5R2 | Animal Meal | Enamel | -0.29 | -0.12 |
| 35127 |  | Animal Meal | Feed | 0.00 | 0.17 |
| 35109 | 2AA6B1 | Day-old-chicks | Bone | 0.02 | 0.06 |
| 35141 | 2AA6B1 | Day-old-chicks | Bone | -0.03 | 0.01 |
| 35121 | 2AA6B1 | Day-old-chicks | Feces | 0.03 | 0.07 |
| 35122 | 2AA6B1 | Day-old-chicks | Hair | -0.12 | -0.08 |
| 35115 | 2AA6B1 | Day-old-chicks | Kidney | -0.82 | -0.78 |
| 35143 | 2AA6B1 | Day-old-chicks | Kidney | -0.84 | -0.80 |
| 35112 | 2AA6B1 | Day-old-chicks | Liver | -1.03 | -0.99 |
| 35142 | 2AA6B1 | Day-old-chicks | Liver | -1.02 | -0.98 |
| 35118 | 2AA6B1 | Day-old-chicks | Muscle | -0.76 | -0.72 |
| 35140 | 2AA6B1 | Day-old-chicks | Plasma | -0.04 | 0.00 |
| 35139 | 2AA6B1 | Day-old-chicks | RBC | -0.08 | -0.04 |
| 35125 |  | Day-old-chicks | Feed | 0.00 | 0.04 |
| 37603 | 1ARA4B1 | Insect Meal | Cem./Den. | -0.26 | -0.02 |
| 35761 | 1ARA4B1 | Insect Meal | Enamel | -0.11 | 0.13 |
| 35106 | 1ARA4B2 | Insect Meal | Bone | 0.30 | 0.54 |
| 37604 | 1ARA4B2 | Insect Meal | Cem./Den. | -0.28 | -0.04 |
| 37623 | 1ARA4B2 | Insect Meal | Enamel | -0.31 | -0.07 |
| 35225 | 1ARA4B2 | Insect Meal | Feces | -0.46 | -0.22 |
| 35229 | 1ARA4B2 | Insect Meal | Hair | 0.02 | 0.26 |
| 35233 | 1ARA4B2 | Insect Meal | Kidney | -0.87 | -0.63 |
| 35237 | 1ARA4B2 | Insect Meal | Liver | -1.17 | -0.93 |
| 35241 | 1ARA4B2 | Insect Meal | Muscle | -0.73 | -0.49 |
| 35253 | 1ARA4B2 | Insect Meal | Plasma | -0.24 | 0.00 |
| 35259 | 1ARA4B2 | Insect Meal | RBC | -0.09 | 0.15 |
| 35338 | 1ARA4B2 | Insect Meal | RBC | -0.11 | 0.13 |
| 37608 | 1ARA4G1 | Insect Meal | Cem./Den. | -0.29 | -0.05 |
| 37624 | 1ARA4G1 | Insect Meal | Enamel | -0.30 | -0.06 |
| 35128 |  | Insect Meal | Feed | 0.00 | 0.24 |
| 35105 | 1ARB1B1 | Lucerne | Bone | 0.53 | 0.59 |
| 37606 | 1ARB1B1 | Lucerne | Cem./Den. | -0.12 | -0.06 |
| 37620 | 1ARB1B1 | Lucerne | Enamel | -0.13 | -0.07 |
| 35119 | 1ARB1B1 | Lucerne | Feces | 0.03 | 0.09 |
| 35249 | 1ARB1B1 | Lucerne | Hair | 0.29 | 0.35 |
| 35113 | 1ARB1B1 | Lucerne | Kidney | -0.62 | -0.56 |
| 35110 | 1ARB1B1 | Lucerne | Liver | -1.00 | -0.94 |
| 35116 | 1ARB1B1 | Lucerne | Muscle | -0.74 | -0.68 |
| 35133 | 1ARB1B1 | Lucerne | Plasma | -0.06 | 0.00 |
| 35132 | 1ARB1B1 | Lucerne | RBC | 0.01 | 0.07 |
| 36027 | 1ARB1B2 | Lucerne | Bone | 0.53 | 0.59 |
| 37602 | 1ARB1B2 | Lucerne | Cem./Den. | -0.17 | -0.11 |
| 37619 | 1ARB1B2 | Lucerne | Enamel | -0.07 | -0.01 |
| 36034 | 1ARB1B2 | Lucerne | Feces | -0.01 | 0.05 |
| 36044 | 1ARB1B2 | Lucerne | Hair | 0.43 | 0.49 |
| 36737 | 1ARB1B2 | Lucerne | Kidney | -0.55 | -0.49 |
| 36744 | 1ARB1B2 | Lucerne | Liver | -0.88 | -0.82 |
| 36750 | 1ARB1B2 | Lucerne | Muscle | -0.59 | -0.53 |
| 36028 | 1ARB1G1 | Lucerne | Bone | 0.58 | 0.64 |
| 37607 | 1ARB1G1 | Lucerne | Cem./Den. | -0.18 | -0.12 |
| 37621 | 1ARB1G1 | Lucerne | Enamel | -0.18 | -0.12 |
| 36035 | 1ARB1G1 | Lucerne | Feces | 0.09 | 0.15 |
| 36045 | 1ARB1G1 | Lucerne | Hair | 0.40 | 0.46 |
| 36738 | 1ARB1G1 | Lucerne | Kidney | -0.62 | -0.56 |
| 36745 | 1ARB1G1 | Lucerne | Liver | -0.98 | -0.92 |
| 36751 | 1ARB1G1 | Lucerne | Muscle | -0.71 | -0.65 |
| 36029 | 1ARB1G2 | Lucerne | Bone | 0.60 | 0.66 |
| 37611 | 1ARB1G2 | Lucerne | Cem./Den. | -0.18 | -0.12 |
| 37622 | 1ARB1G2 | Lucerne | Enamel | -0.20 | -0.14 |
| 36036 | 1ARB1G2 | Lucerne | Feces | -0.02 | 0.04 |
| 36046 | 1ARB1G2 | Lucerne | Hair | 0.38 | 0.44 |
| 36739 | 1ARB1G2 | Lucerne | Kidney | -0.61 | -0.55 |
| 36746 | 1ARB1G2 | Lucerne | Liver | -0.94 | -0.88 |
| 36752 | 1ARB1G2 | Lucerne | Muscle | -0.59 | -0.53 |
| 36030 | 1ARB1R1 | Lucerne | Bone | 0.55 | 0.61 |
| 36042 | 1ARB1R1 | Lucerne | Feces | -0.04 | 0.02 |
| 36047 | 1ARB1R1 | Lucerne | Hair | 0.43 | 0.49 |
| 36740 | 1ARB1R1 | Lucerne | Kidney | -0.55 | -0.49 |
| 36747 | 1ARB1R1 | Lucerne | Liver | -0.99 | -0.93 |
| 36753 | 1ARB1R1 | Lucerne | Muscle | -0.65 | -0.59 |
| 36031 | 1ARB1R2 | Lucerne | Bone | 0.50 | 0.56 |
| 37612 | 1ARB1R2 | Lucerne | Cem./Den. | -0.16 | -0.10 |
| 36043 | 1ARB1R2 | Lucerne | Feces | 0.04 | 0.10 |
| 36048 | 1ARB1R2 | Lucerne | Hair | 0.47 | 0.53 |
| 36741 | 1ARB1R2 | Lucerne | Kidney | -0.59 | -0.53 |
| 36748 | 1ARB1R2 | Lucerne | Liver | -0.96 | -0.90 |
| 36754 | 1ARB1R2 | Lucerne | Muscle | -0.50 | -0.44 |
| 35124 |  | Lucerne | Feed | 0.05 | 0.11 |
| 36040 |  | Lucerne | Feed | -0.02 | 0.04 |
| 36041 |  | Lucerne | Feed | -0.03 | 0.03 |
| 36026 | 1BRA1B1 | Supplier | Bone | 0.04 | 0.10 |
| 37616 | 1BRA1B1 | Supplier |  | -0.18 | -0.12 |
| 37615 | 1BRA1B1 | Supplier | Enamel | -0.13 | -0.07 |
| 36033 | 1BRA1B1 | Supplier | Feces | 0.10 | 0.16 |
| 36736 | 1BRA1B1 | Supplier | Kidney | -0.43 | -0.37 |
| 36743 | 1BRA1B1 | Supplier | Liver | -0.78 | -0.72 |
| 36749 | 1BRA1B1 | Supplier | Muscle | -0.52 | -0.46 |
| 36025 | 1BRC1B1 | Supplier | Bone | -0.03 | 0.03 |
| 37618 | 1BRC1B1 | Supplier | Cem./Den. | -0.12 | -0.06 |
| 37617 | 1BRC1B1 | Supplier | Enamel | -0.15 | -0.09 |
| 36032 | 1BRC1B1 | Supplier | Feces | -0.12 | -0.06 |
| 36735 | 1BRC1B1 | Supplier | Kidney | -0.58 | -0.52 |
| 36742 | 1BRC1B1 | Supplier | Liver | -0.93 | -0.87 |
| 35223 | 1BRC4B1 | Supplier | Bone | -0.15 | -0.09 |
| 35227 | 1BRC4B1 | Supplier | Feces | 0.08 | 0.14 |
| 35231 | 1BRC4B1 | Supplier | Hair | -0.34 | -0.28 |
| 35235 | 1BRC4B1 | Supplier | Kidney | -0.61 | -0.55 |
| 35239 | 1BRC4B1 | Supplier | Liver | -0.98 | -0.92 |
| 35243 | 1BRC4B1 | Supplier | Muscle | -0.73 | -0.67 |
| 35255 | 1BRC4B1 | Supplier | Plasma | -0.06 | 0.00 |
| 35257 | 1BRC4B1 | Supplier | RBC | 0.04 | 0.10 |
| 35336 | 1BRC4B1 | Supplier | RBC | 0.02 | 0.08 |
| 35340 | 1BRC4B1 | Supplier | RBC | 0.08 | 0.14 |
| 35126 |  | Supplier | Feed | -0.15 | -0.09 |
| 35248 |  | Supplier | Feed | 0.01 | 0.07 |
| 36037 |  | Supplier | Feed | 0.08 | 0.14 |
| 36038 |  | Supplier | Feed | 0.06 | 0.12 |
| 36039 |  | Supplier | Feed | 0.00 | 0.06 |
| 35224 | 2AA1G1 | Vegetable Mix | Bone | -0.08 | 0.04 |
| 35228 | 2AA1G1 | Vegetable Mix | Feces | -0.20 | -0.08 |
| 35232 | 2AA1G1 | Vegetable Mix | Hair | -0.22 | -0.10 |
| 35250 | 2AA1G1 | Vegetable Mix | Hair | -0.27 | -0.15 |
| 35236 | 2AA1G1 | Vegetable Mix | Kidney | -0.71 | -0.59 |
| 35240 | 2AA1G1 | Vegetable Mix | Liver | -1.07 | -0.95 |
| 35244 | 2AA1G1 | Vegetable Mix | Muscle | -0.88 | -0.76 |
| 35256 | 2AA1G1 | Vegetable Mix | Plasma | -0.12 | 0.00 |
| 35260 | 2AA1G1 | Vegetable Mix | RBC | -0.09 | 0.03 |
| 35339 | 2AA1G1 | Vegetable Mix | RBC | -0.12 | 0.00 |
| 35341 | 2AA1G1 | Vegetable Mix | RBC | -0.09 | 0.03 |
| 35245 |  | Vegetable Mix | Feed | 0.00 | 0.12 |

**Table S5. The Δ^66^Zn values relatives to the mean of the feed (Δ^66^Zn_diet_), and plasma (Δ^66^Zn_plasma_), with corresponding laboratory internal S-EVA number, original sample number (i.e., rat specimens’ ID), dietary group, and sample type (organ, biofluids, excreta, and feed). Red blood cells and cementum/dentine are respectively denoted by abbreviation RBC and Cem./Den. Abbreviations: S-EVA = Stable isotope-Evolutionary Anthropology (the numbering system used in the Human Evolution department of the Max Planck Institute for Evolution Anthropology, Leipzig, for every sample analyzed for isotopic analysis).**


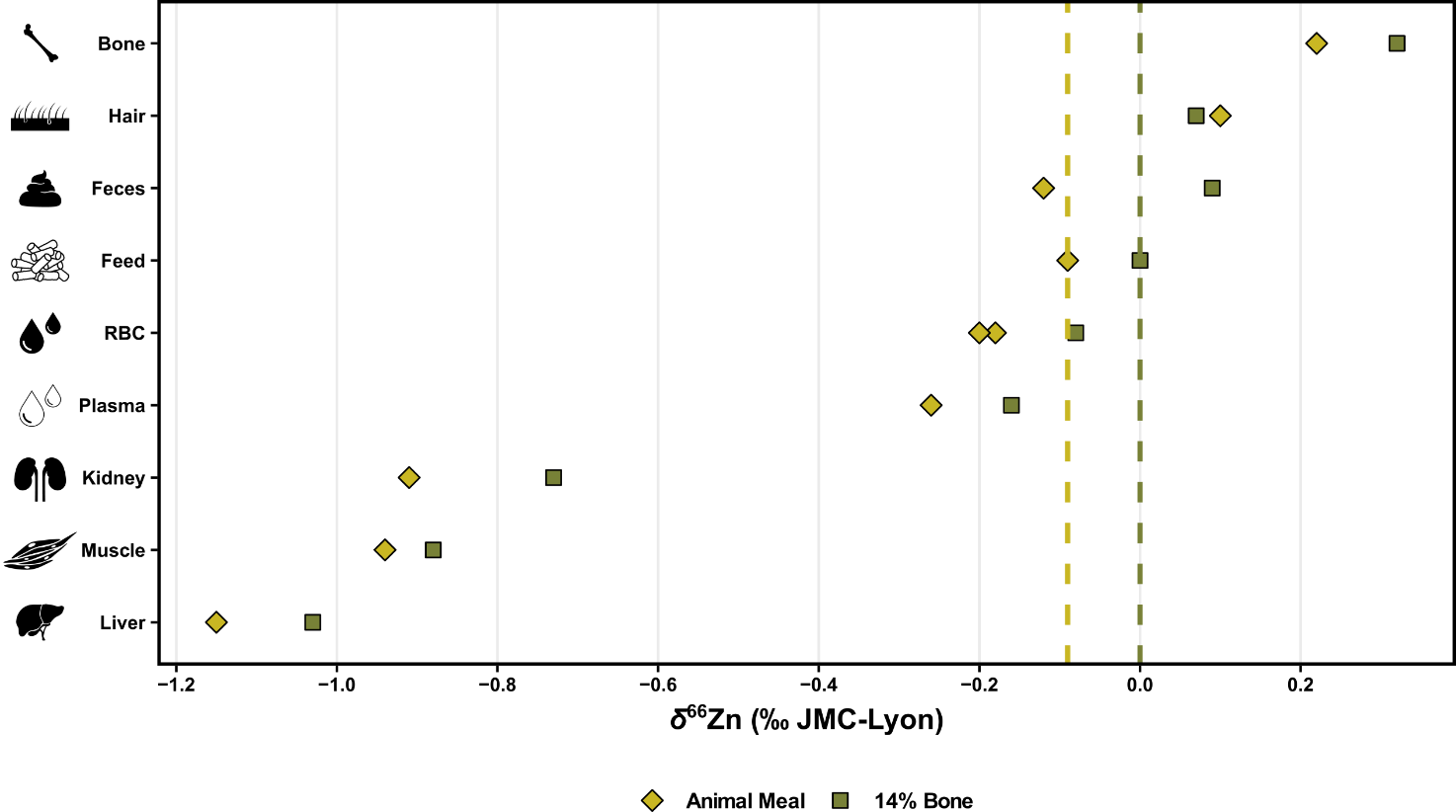


**Figure S1.** The zinc isotope compositions (‰, relative to the JMC-Lyon zinc isotope standard) of the liver, muscle, kidney, plasma, red blood cells (RBC), feces, hair, and bone, and respective feeds for the animal meal pelleted diet (light green-yellow diamond) and the 14% Bone pelleted diet (green square). Note that all tissue and excreta *δ*^66^Zn values associated with the 14% Bone pelleted diet are reflecting the ^66^Zn-enrichment of this feed compared to the animal meal pellet and thus are all accordingly exhibiting higher *δ*^66^Zn values. This demonstrates that even small zinc isotope differences of 0.1 ‰ in the diet are reflected in consumer tissues. The dashed lines correspond to the *δ*^66^Zn values of the diet supplied to the animals for each diet.


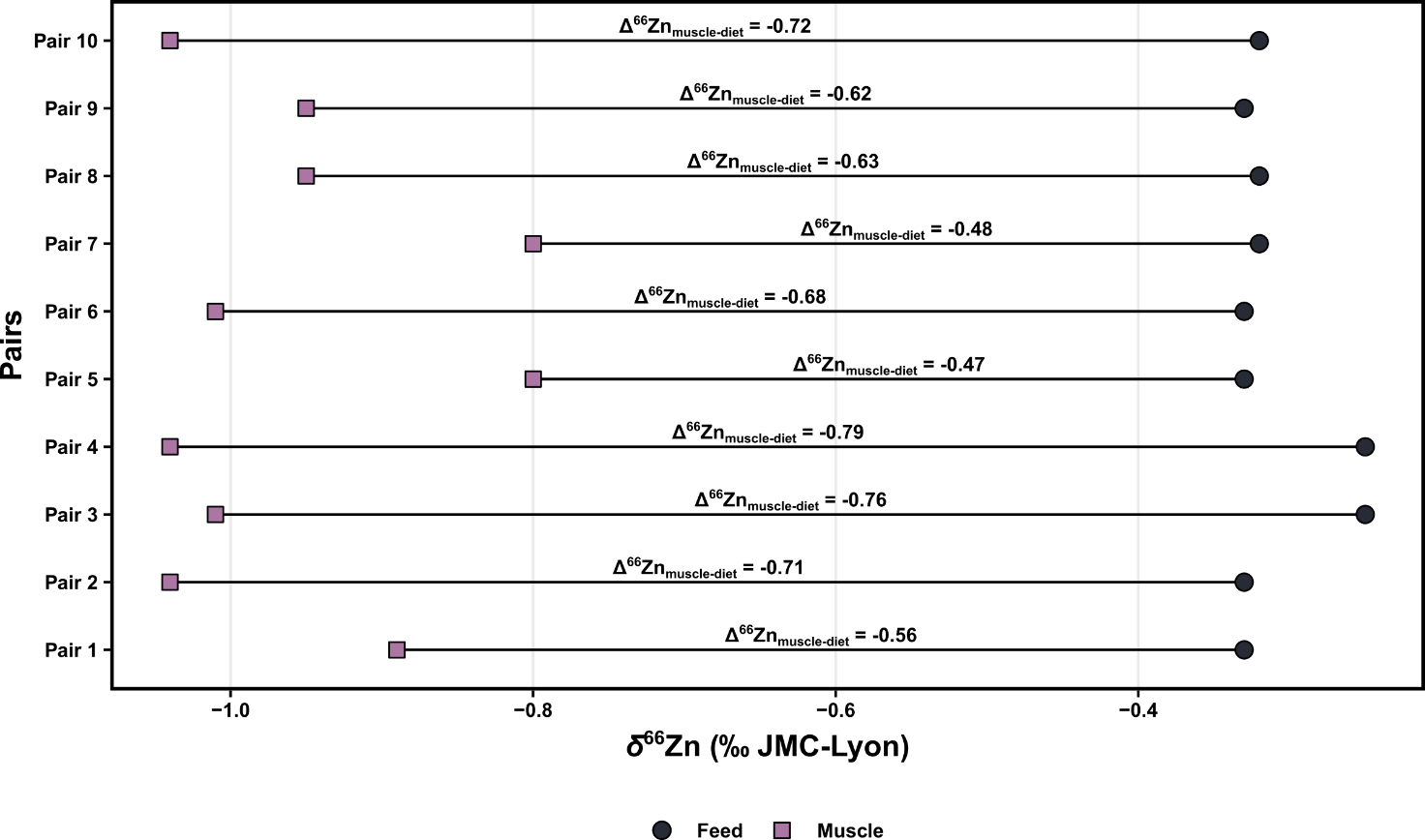


**Figure S2. The zinc isotope compositions (‰, relative to the JMC-Lyon zinc isotope standard) of randomly-selected pairs of *δ*^66^Zn values of feed (*n* = 3) and muscle tissues from the pelleted lucerne diet (*n* = 6), and the respective Δ^66^Zn_muscle-diet_ value of each pairing, here ranging from -0.79 to -0.47 ‰.**


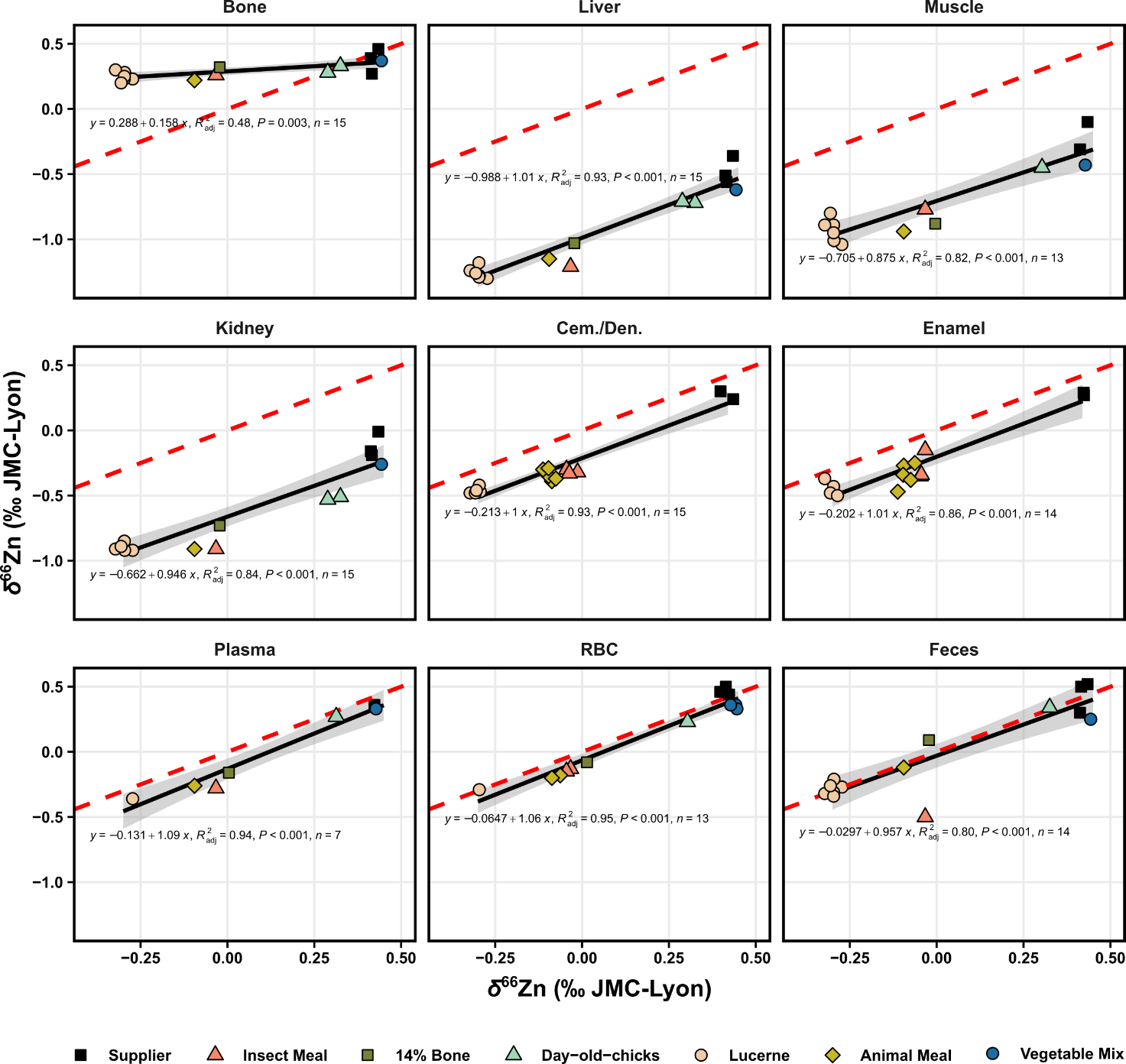


**Figure S3. The zinc isotope compositions (‰, relative to the JMC-Lyon zinc isotope standard) of the liver, muscle, kidney, plasma, red blood cells (RBC), feces, bone, enamel, cementum/dentine (Cem./Den.) relative to the zinc isotope compositions of their respective feeds (pelleted supplier’s diet, pelleted animal meal diet, pelleted insect meal diet, pelleted lucerne meal, pelleted animal meal diet with a 14% bone-meal supplement, vegetable mix diet, and a day-old-chick diet). The dashed red line correspond to a theoretical 1:1, while the black line represent the actual relationship between the zinc isotope compositions of the tissues and the diet, which are systematically in agreement with the slope of theoretical one, except for bone. The shaded area corresponds to 95% confidence intervals of the observed compositions.**

# ****Supplementary Material-2: Sweep CI fits****

## **Defining Zn cycle series**

Because the nominal values of some Zn fluxes and Zn isotope fractionations in rats remain unknown or poorly constrained, we simulated the evolutions of Zn isotope distributions in rats by sweeping these parameters over wide ranges of values prior to running either Model 1 or 2. For this purpose, we used the sweep.final_nD and fit.final_space functions of the isobxr R package to estimate the best parameter values allowing to reproduce the steady-state Zn isotope compositions (within confidence intervals) reported for the key reservoirs of the organism [1].

The Zn isotope compositions (used to calculate α_i-j_; see **Table 3**) and associated data (e.g., feed's zinc concentration) measured for the supplier rats were used as the reference steady-state status of the system, as supplier rats are assumed to be at isotopic equilibrium with their diets since their last dietary switch occurred at weaning from breastmilk. Box sizes (i.e., mass of Zn in each box, **Table 1**) and initial Zn fluxes (**Figure 1**) were taken from House and Wastney (1997), and the net absorption was set to 305 µg/d based on House and Wastney (1997) and Høyer and Weismann (1979) [2,3]. Lastly, the intake was set to 1000 µg/d, calculated from the average zinc concentration (rounded to 100 µg/g) in the supplier's diet and the daily food (15 g per day); the fractional absorption thus corresponds to around ca. 30% of the daily intake.

**
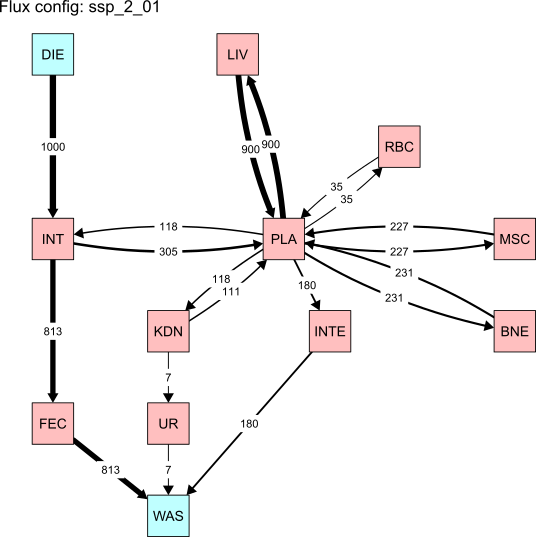
**

**Figure 1.** Initial flux structure (in µg/d) of the Zn cycle prior to the sweep space parameters, between compartments: diet (DIE), intestine (INT), plasma (PLA), erythrocytes (i.e., red blood cells; RBC), liver (LIV), muscles (MSC), bones (BNE), kidneys (KDN), hair (considered here as integument; INTE), urine (UR), feces (FEC), and waste (WAS).

| **Compartment** | **Zinc content in compartment (μg)** |
| --- | --- |
| **Diet** | Infinite |
| **Intestine** | 1000 |
| **Plasma** | 32 |
| **Liver** | 378 |
| **Red Blood Cells** | 320 |
| **Muscle** | 1960 |
| **Bone** | 3212 |
| **Kidney** | 63 |
| **Integument** | 2787 |
| **Feces** | 813 |
| **Urine** | 7 |
| **Waste** | Infinite |

**Table 1.** List of masses (μg) of each compartment used in the current study for a rat of 370 g, taken from House & Wastney (1997) [2].

### **Space of parameters**

#### **Sweeping of the endogenous vs. integument balance**

Varying flux configurations were swept to encompass the various Zn cycles in mammals, as reported in humans and rats. The fluxes that are the most critical in determining the steady-state isotope compositions of an organism are those responsible for the exchange of the element with the environment. In the case of Zn in mammals, the environment-organism exchanges occur upon (1) intestinal absorption (INT->PLA), (2) endogenous losses (PLA->INT), (3) urinary losses (KDN->UR), and (4) integumentary losses (PLA->INTEG). The urinary losses are well constrained and limited in rats of 7 µg/d Zn, accounting for about 2% of intestinally absorbed Zn [2,3]. As detailed above, intestinal absorption is estimated to lie around 300 µg/d.

However, the relative importance of integumentary and endogenous losses is somewhat uncertain, as there is limited yet contrasting data in rats. In rats, some studies suggest a strong loss of Zn in the integument of the order of 180 µg/d, corresponding to ca. 59% of absorbed Zn [2]. In contrast, the rate of zinc loss through the skin in humans is about 250 to 300 µg/d for an absorption of ca. 4000 µg/d, thus representing a relative integumentary Zn loss of only 6.5-7.5% of absorbed Zn [4,5].

Sets of sweep space parameters (ssp; **Table 2**) were thus established to evaluate varying degrees of integumentary Zn loss, whereby ssp_2_01 corresponds to the highest integument flux (as suggested by House and Wastney, 1997) and ssp_2_11 to low integument one (as suggested for humans by Jaouen et al., 2019) [2,5]. The relative proportion of endogenous losses was accordingly adjusted to ensure a Zn balance in line with a fractional absorption of around 30% of dietary intake, as discussed above. The maximal relaxation times (t_relax_) were estimated for these Zn cycles to assess the minimum duration required to reach the steady-state. These maximum t_relax_ values range from 33 to 696 days between ssp_2_01 and ssp_2_11. We usually consider that the steady-state is reached after more than 5*t_relax_. Therefore, we set the total run durations to 10,000 days for all simulations aiming at relaxing the system to steady-state.

| **flux list name** | **Absorption** | | **Endogenous losses** | | **Integumentary losses** | | **Urinary losses** | |
| --- | --- | --- | --- | --- | --- | --- | --- | --- |
|  | **Apparent frac. abs.** | **True frac. abs.** | **Relative loss** | **Flux PLA-INT** | **Relative loss** | **Flux PLA-INTEG** | **Relative loss** | **Flux KDN-UR** |
|  | *%* | *%* | *%* | *µg/d* | *%* | *µg/d* | *%* | *µg/d* |
| **ssp_2_01** | 19% | 31% | 39% | 118 | 59% | 180 | 2% | 7 |
| **ssp_2_02** | 15% | 31% | 52% | 132 | 46% | 166 | 2% | 7 |
| **ssp_2_03** | 11% | 31% | 65% | 146 | 33% | 152 | 2% | 7 |
| **ssp_2_04** | 7% | 31% | 78% | 160 | 20% | 138 | 2% | 7 |
| **ssp_2_05** | 5% | 31% | 85% | 174 | 13% | 124 | 2% | 7 |
| **ssp_2_06** | 4% | 31% | 88% | 188 | 10% | 110 | 2% | 7 |
| **ssp_2_07** | 3% | 31% | 91% | 202 | 7% | 96 | 2% | 7 |
| **ssp_2_08** | 2% | 31% | 93% | 216 | 5% | 82 | 2% | 7 |
| **ssp_2_09** | 2% | 31% | 94% | 230 | 3% | 68 | 2% | 7 |
| **ssp_2_10** | 1% | 31% | 96% | 244 | 2% | 54 | 2% | 7 |
| **ssp_2_11** | 1% | 31% | 96% | 258 | 1% | 40 | 2% | 7 |

**Table 2.** Sets of sweep space parameters that were used to explore Zn losses in the environment-organism exchanges in a rat: (1) endogenous losses (PLA->INT), (2) integumentary losses (PLA->INTEG), and (3) urinary losses (KDN->UR). Each flux configuration (µg/d) is then associated with a relative loss (%) and the overall apparent absorption (%) of the organism. The apparent fractional absorption refers to the observed difference between dietary intake and fecal losses normalized to dietary intake: 100*(1-*F_(INT>FEC)_⁄F_(DIET>INT)_*) where F refers to the Zn flux in µg/d. The true fractional absorption refers to the ratio between effectively absorbed Zn and dietary intake: 100*(*F_(INT>PLA)_⁄F_(DIET>INT)_*).

#### **Sweeping of isotope fractionation coefficients**

In addition to the sweeping of fluxes relative to the endogenous vs. integument balance, sets of isotope fractionation coefficients were also explored. Notably, the fractionation upon intestinal absorption (α_INT-PLA_) and endogenous losses (α_PLA-INT_) were set to vary between 0.9997 and 1.0003 (corresponding to -0.3 and +0.3 ‰), owing to the lack of precise basal estimate measured yet. Similarly, no precise basal estimate for isotope fractionation upon urinary losses (α_KDN-UR_) has been measured yet, and was thus here swept between 1.0000 and 1.0010 (0 and 1 ‰), in favor of heavy isotopes as seen in humans [6]. Lastly, fractionation upon integument transport (α_PLA-INTEG_) was set to vary between 0.9995 and 1.0000 (-0.50 and 0 ‰), as integument values found in supplier rat (hair) are around -0.30 ‰ lower than plasma, but the fractionation is otherwise undescribed yet.

| **FROM** *( i )* | **TO**  *( j )* | **Coefficient  α_i-j_** | | |  | **Corresponding  Δ_i-j_ (‰)** | | | **Parameter sweeping** |
| --- | --- | --- | --- | --- | --- | --- | --- | --- | --- |
| DIET | INT | 1.00000 | | |  | 0.00 | | | constant |
| INT | FEC | 1.00000 | | |  | 0.00 | | | constant |
| PLA | LIV | 0.99958 | | |  | -0.42 | | | constant |
| PLA | RBC | 1.00005 | | |  | 0.05 | | | constant |
| PLA | MSC | 0.99972 | | |  | -0.28 | | | constant |
| PLA | BNE | 1.00001 | | |  | 0.01 | | | constant |
| PLA | KDN | 1.00000 | | |  | 0.00 | | | constant |
| LIV | PLA | 1.00042 | | |  | 0.42 | | | constant |
| RBC | PLA | 0.99995 | | |  | -0.05 | | | constant |
| MSC | PLA | 1.00028 | | |  | 0.28 | | | constant |
| BNE | PLA | 0.99999 | | |  | -0.01 | | | constant |
| KDN | PLA | 1.00000 | | |  | 0.00 | | | constant |
| INT | PLA | 0.99970 | to | 1.00030 |  | -0.30 | to | 0.30 | swept over 10 values |
| PLA | INT | 0.99970 | to | 1.00030 |  | -0.30 | to | 0.30 | swept over 10 values |
| KDN | UR | 1.00000 | to | 1.00100 |  | 0.00 | to | 1.00 | swept over 10 values |
| PLA | INTEG | 0.99950 | to | 1.00000 |  | -0.50 | to | 0.00 | swept over 10 values |

**Table 3.** List of fractionation coefficients between compartments used in the current study. Fractionation coefficient are calculated from *δ*^66^Zn values from the current study or deduced.

### **CI-fit**

#### **Principle of the CI-fit approach**

The sweeping of the space of parameters was performed using the sweep.final_nD function of isobxr R package [1]. The space of parameters entails 11 distinct flux configurations (**Figure 1** and **Table 2**) as well as 4 isotope fractionation coefficients (**Table 3**), with each 10 values evenly distributed across the targeted ranges. The exhaustive sweeping produced as a result 110,000 simulations, corresponding to all combinations of parameter values. For a set of key reservoirs, this method allows to find all the combinations of parameter values allowing to produce steady-state isotope compositions falling within the observed confidence intervals of all reservoirs of interest. To this end, we use the fit.final_space function of isobxr R package.

As mentioned before, the Zn isotope compositions measured in the supplier rats were used as references to compare the swept space of parameters with observed compositions of rats assumed to be at equilibrium with their diets, given that their last dietary switch occurred at weaning from breast milk. The observed confidence intervals (obs.CI) were defined as 2SE and, in the case of boxes with a single observation (PLA and INTEG), the obs.CI were conservatively set to 0.2 ‰, corresponding to 4*maximized external reproducibility (roughly 0.05 ‰ on repeated measurement of standards) and also to the maximum of estimated 2SE on other boxes with *n* ≥ 2 individuals (**Table 4**).

| **Organ** | **Observations** | | | | **Defining obs.CI** | | |
| --- | --- | --- | --- | --- | --- | --- | --- |
|  | **Mean *δ*^66^Zn** | **∆^66^Zn_diet_** | **SD** | ***n*** | **CI.T** | **2SE 2SD/sqrt(*n*)** | **obs.CI** |
| **Bone** | 0.37 | -0.05 | 0.1 | 3 | 0.25 | 0.12 | 0.12 |
| **Feces** | 0.44 | 0.02 | 0.12 | 3 | 0.30 | 0.14 | 0.14 |
| **Feed*** | 0.42 | 0.00 | 0.09 | 5 | 0.11 | 0.08 | 0.08 |
| **Hair** | 0.08 | -0.34 | - | 1 | - | - | 0.20 |
| **Kidney*** | -0.12 | -0.54 | 0.1 | 3 | 0.25 | 0.12 | 0.12 |
| **Liver** | -0.48 | -0.90 | 0.1 | 3 | 0.25 | 0.12 | 0.12 |
| **Muscle** | -0.21 | -0.62 | 0.15 | 2 | 1.35 | 0.21 | 0.21 |
| **Plasma** | 0.36 | -0.06 | - | 1 | - | - | 0.20 |
| **RBC** | 0.47 | 0.05 | 0.03 | 3 | 0.07 | 0.03 | 0.03 |

**Table 4.** List of *δ*^66^Zn values per tissue, biofluid, and excreta, from the rat specimens fed only their supplier's feed, used to establish confidence interval (CI) used for the sweeping of the space of parameters. *Refers to boxes that were excluded from the CI-fits.

Lastly, it is important to mention that the kidney was excluded from simulation vs. observation fitting; the current design treats it as an interface and thus does not aim to reproduce its true isotopic composition. Moreover, it represents a too complex organ to be modelled effectively in the model's current form and thus is beyond the scope of this study.

#### **Results of the confidence-interval fittings**

##### *Fit_0: Fitting over all parameter values*

Overall, this CI-fit shows a very good agreement between observed and predicted steady-state (**Figure 2A**). The density distributions of the fitted fractionation coefficients are not markedly constraining with the exception of the PLA-INTEG fractionation coefficient, with a best fit value of -0.28 ± 0.12 ‰ (expressed as a Δ_PLA-INTEG_). Finally, all flux configurations (ssp_2_01 to ssp_2_11) are equally represented in the CI-fits.

##### *Effect of renal isotope fractionation / fit_1 to fit_3*

We assume here two extreme hypotheses with (1) an absence of renal fractionation (Δ_PLA-INTEG_ = 0 ‰, fit_1) and (2) a constant and strong renal fractionation inducing a preferential loss of urinary Zn enriched by 1 ‰ (fit _2). Both corresponding CI-fits show that all the fitted simulations predict compositions comparable in both hypotheses (with differences lower than 0.01 ‰), irrespective of the extant of renal fractionation, being of 0 or 1 ‰ (**Figure 2A**). This is true for all reservoirs other than kidney and urine, which are directly affected, as expected. The density distributions of the corresponding fitted values of all parameters other than the renal fractionation coefficient also remain unchanged (**Figure 2B**).

In other words, the extent of the renal isotope fractionation has no importance in determining the steady-state isotope compositions of the organism itself. We thus set thereafter the renal isotope fractionation at 0.44 ‰ (α_KDN-UR_ = 1.00044). This amplitude is comparable to the average offset in *δ*^66^Zn between urine and plasma previously reported in humans [6].


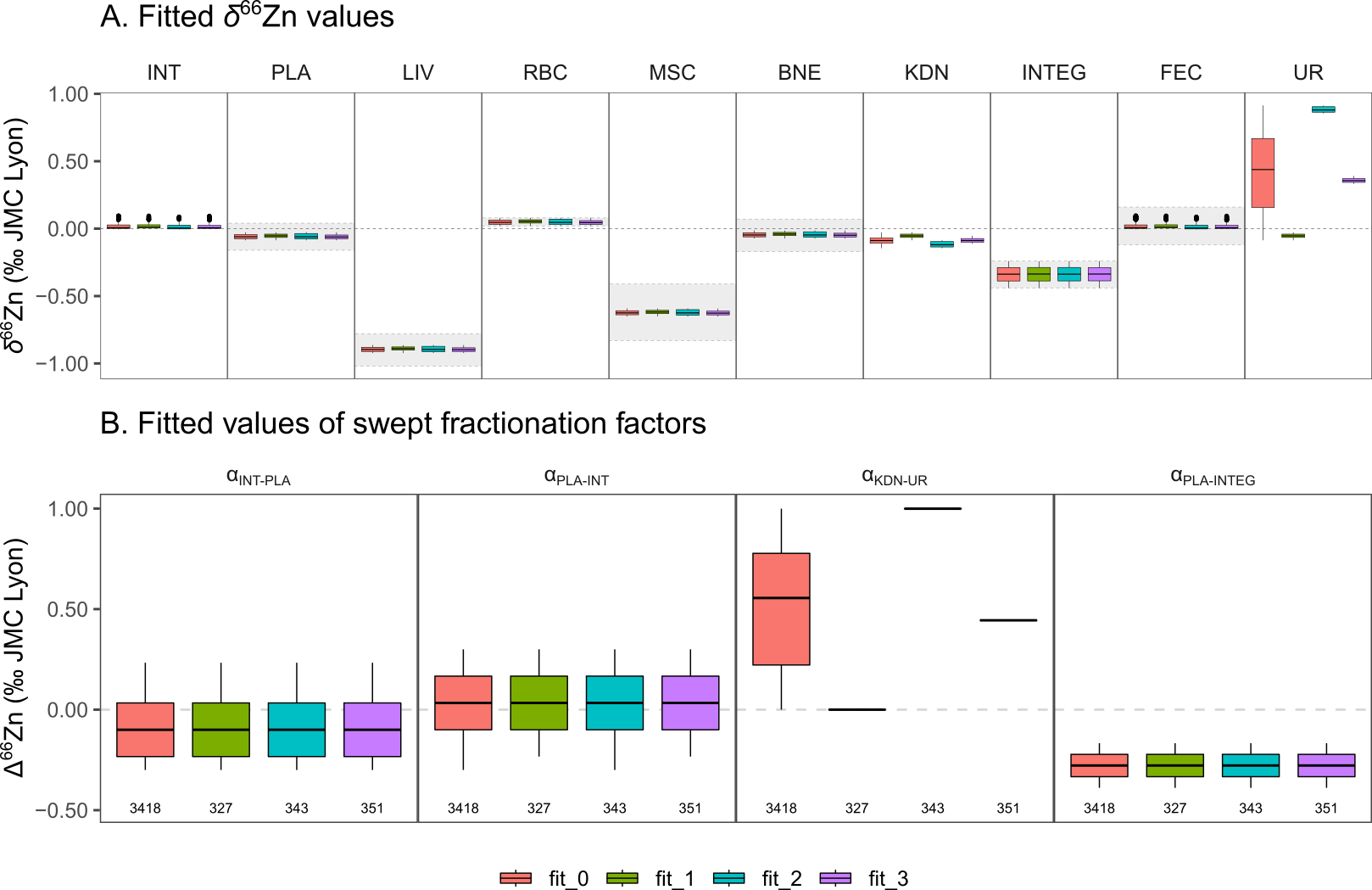


**Figure 2.** **A**. Distributions of fitted δ^66^Zn values in all boxes: intestine (INT), plasma (PLA), liver (LIV), red blood cells (RBC), muscle (MSC), bone (BNE), kidney (KDN), integument (INTEG; i.e., in this case, hair), feces (FEC), and urine (UR). Shaded area corresponds to confidence intervals of the observed compositions used for the CI-fits. **B**. Distributions of fitted swept fractionation coefficients, plotted as ∆ values expressed in ‰. The number of fitted simulations (out of 110 000 simulations) is shown at the bottom.

##### *Fit_4: Effect of fractionation upon intestinal absorption, endogenous losses, and integument transport*

We then determine the sets of CI-fitted values of the other swept fractionation coefficients (α_INT-PLA_, α_PLA-INT_, α_PLA-INTEG_) for each flux configuration (from ssp_2_01 to ssp_2_11). As a result, each flux configuration is associated with a combination of fractionation coefficient values allowing the simulation of isotope compositions falling within the confidence intervals of the key reservoirs. The distributions of the predicted isotope compositions and the CI-fitted fractionation coefficients are shown in **Figure 3**.


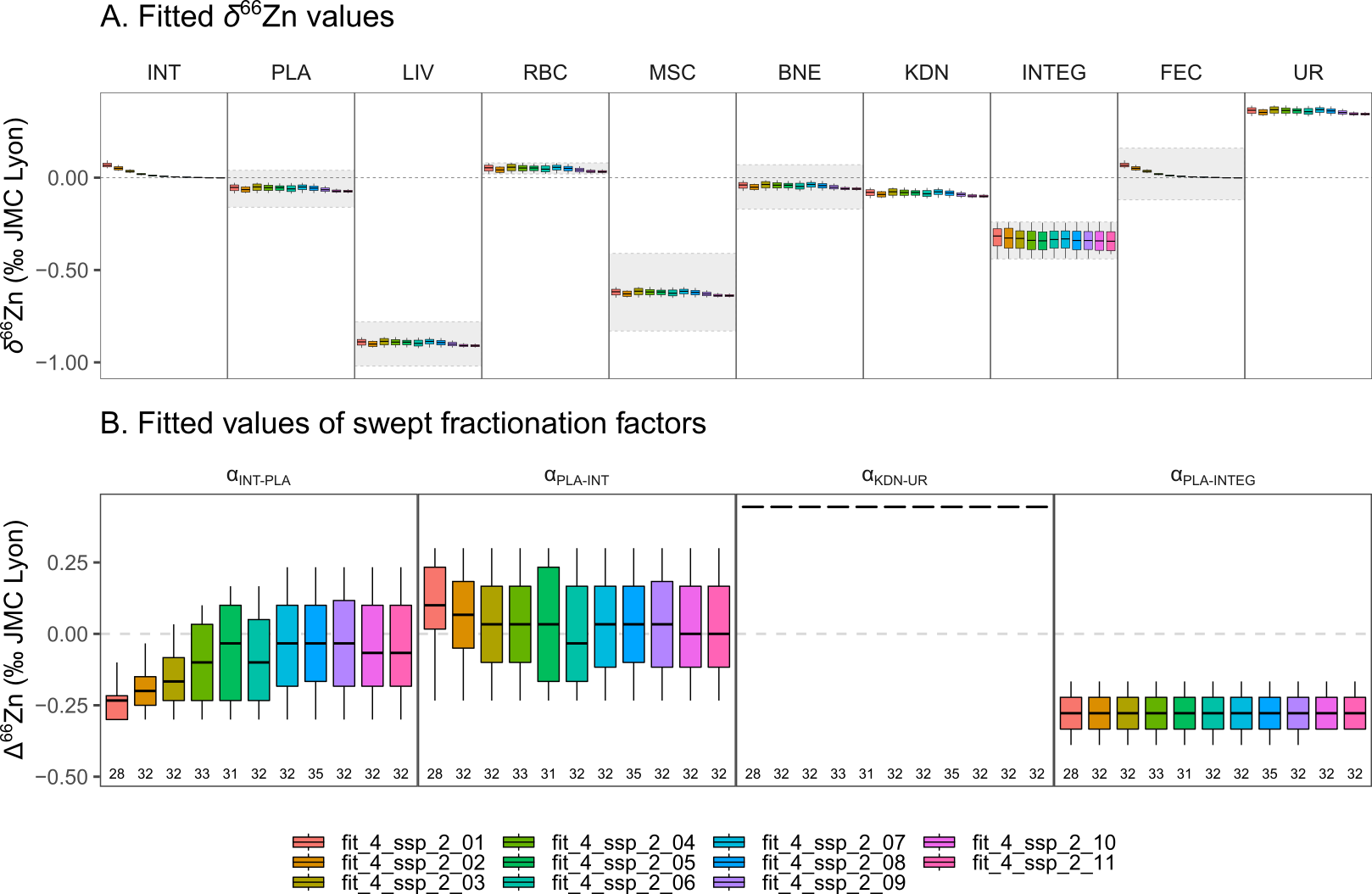


**Figure 3.** **A.** Distributions of fitted δ^66^Zn values in all boxes: intestine (INT), plasma (PLA), liver (LIV), red blood cells (RBC), muscle (MSC), bone (BNE), kidney (KDN), integument (INTEG; i.e., in this case, hair), feces (FEC), and urine (UR). Shaded area corresponds to confidence intervals of the observed compositions. **B.** Distributions of fitted swept fractionation coefficients, plotted as ∆ values expressed in ‰. The number of fitted simulations is shown at the bottom (out of 110 000 simulations).

The CI-fitting of each flux configuration successfully identifies a set of parameter values that produce *δ*^66^Zn predictions falling within the observed confidence intervals. Within those confidence intervals, the *δ*^66^Zn predictions are poorly sensitive to the considered flux configuration. The only obvious trend is observed in feces, which are ^66^Zn enriched for high integument/low endogenous loss ratios. This is explained by the fact that the endogenous losses are hence representing a higher proportion of the Zn fecal losses.

## Supplier – lucerne diet change with varying integument *t_1/2_*

We use here all the fitted sets of parameter values for each flux configuration (ssp_2_01 to ssp_2_11 with varying integumentary/endogenous loss balance) to assess the effects of the residence time of Zn in integument on the isotope compositions of the key reservoirs of the rats after a diet switch to the lucerne diet. These individuals, fed for 59 days on the lucerne pellets are expected to display varying extents of diet-body isotope equilibration depending on the box considered.


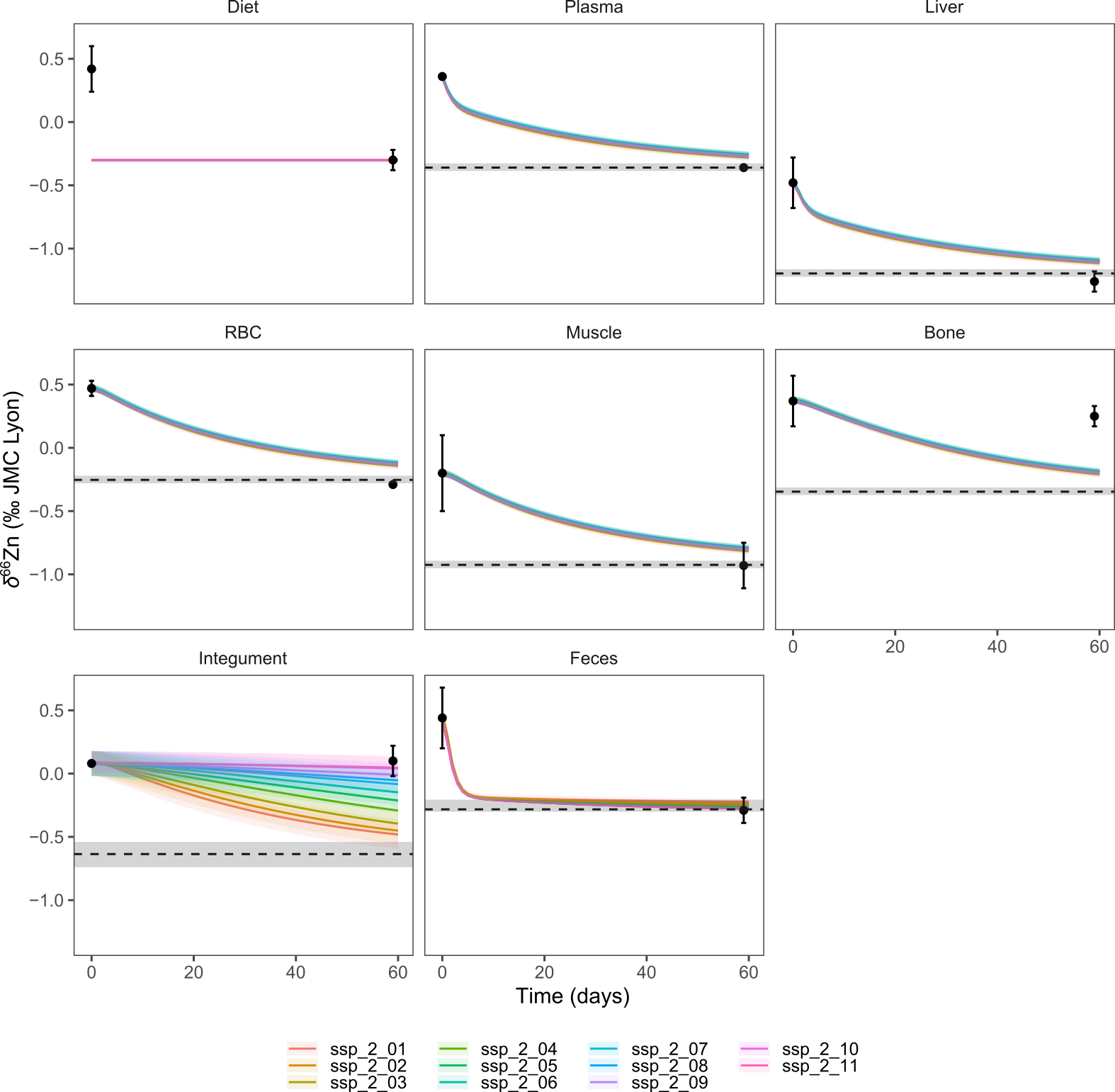


**Figure 4.** Predicted evolution of δ^66^Zn (‰ JMC-Lyon) in each box following a dietary transition from a steady-state organism at equilibrium with the supplier diet to a lucerne pellet diet. The dashed lines with a shaded area correspond to the predicted steady-state isotope composition when the organism is fully equilibrated with the second diet (lucerne pellets here). The black circles correspond to the average values of the observed δ^66^Zn values (error bars are 2SD). The time axis corresponds to the time (in days) that elapsed since the start of the experiment (i.e., the introduction of the second diet). Each curve and shaded area represent the average and full extent of compositions predicted by the sets of fitted parameter values determined for each flux configuration (see fit_4 described above and illustrated in **Figure 3**).

**Figure 4** shows the prediction of the evolution of the organism isotope compositions following a diet switch from the supplier diet to the lucerne diet, using the fitted parameters determined using the rats at equilibrium with the supplier diet only. We observe that irrespective of the assumed flux configuration, the isotope compositions of plasma, liver, red blood cells, muscle, and feces predicted after 59 days are in very good agreement with the *δ*^66^Zn values measured in the rats fed on the lucerne pellets for the same amount of time. Overall, the isotope compositions in those reservoirs have reached full isotopic equilibrium with the new diet. However, the bone appears to remain out of equilibrium in all cases and in poor agreement with the predicted evolution.

The predicted evolution of the zinc isotope composition of the integument is strongly related to the flux configuration, which are characterized by varying PLA-INTEG fluxes and hence varying Zn residence time in integument.


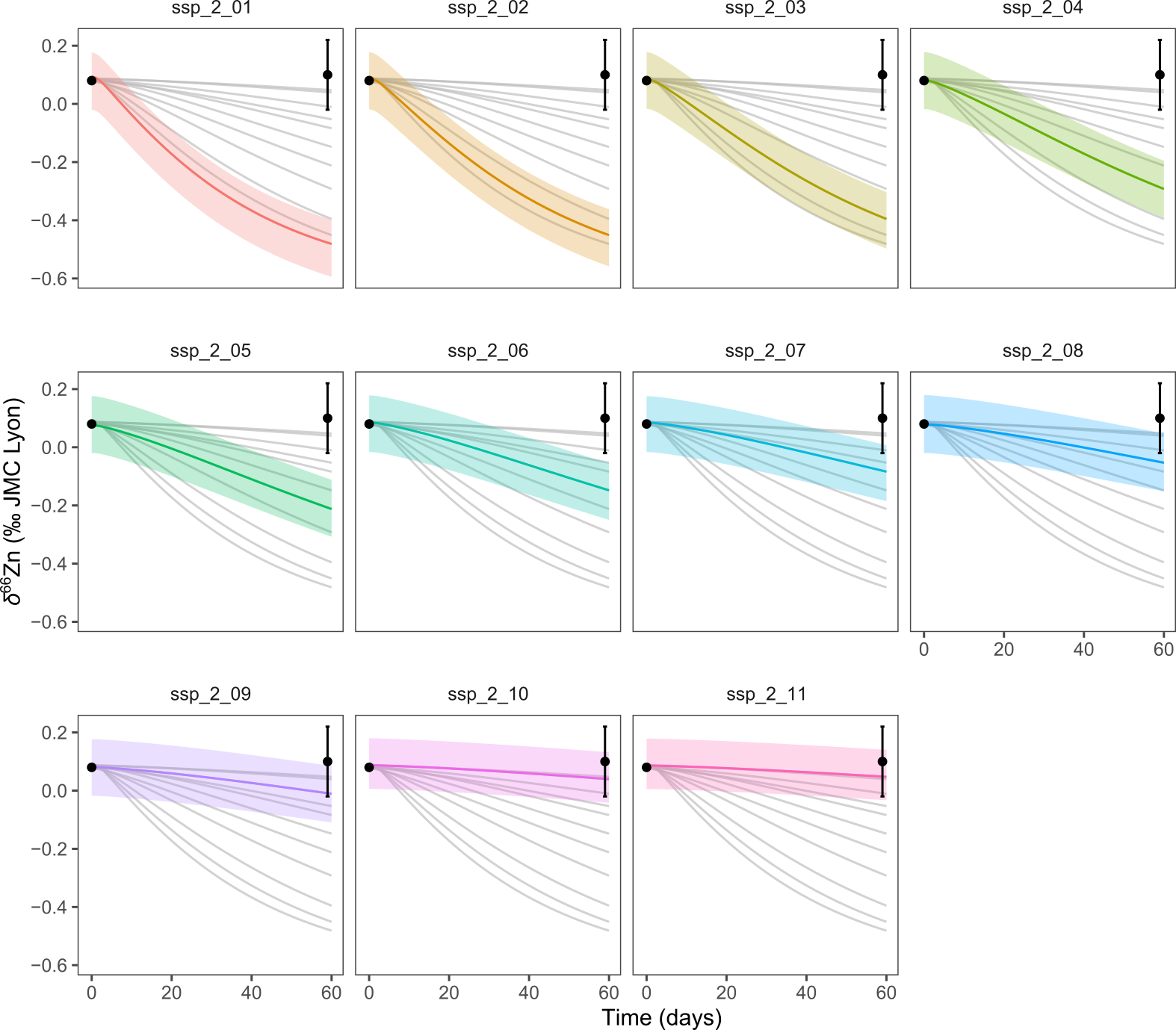


**Figure 5.** Predicted evolution of δ^66^Zn (‰ JMC-Lyon) in hair following a dietary transition from a steady-state organism at equilibrium with the supplier diet to a lucerne pellet diet, whereby different PLA-INTEG fluxes (from 4 to 180 μg/day) are modeled. The colored lines shown with the shaded area correspond to the predicted steady-state isotope composition when the organism is fully equilibrated with the second diet (lucerne pellets here), whereby the grey lines correspond to the prediction using the other fluxes. The black circles correspond to the average values of the observed δ^66^Zn values (error bars are 2SD). The time axis corresponds to the time (in days) that elapsed since the start of the experiment (i.e., the introduction of the second diet). Each curve and shaded area represent the average and full extent of compositions predicted by the sets of fitted parameter values determined for each flux configuration (see fit_4 described above and illustrated in **Figure 3**).

The best integument fit is found in flux configuration ssp_2_11, corresponding to a low integument loss/high endogenous loss comparable to what was described in humans. The *t_1/2_* in integument (hair in the current case) is thus rather in the order of 700 days, not 70 days, as initially assumed in House and Wastney (1997) [2]. We thereafter use the ssp_2_11 flux configuration, shown below, for subsequent modeling.


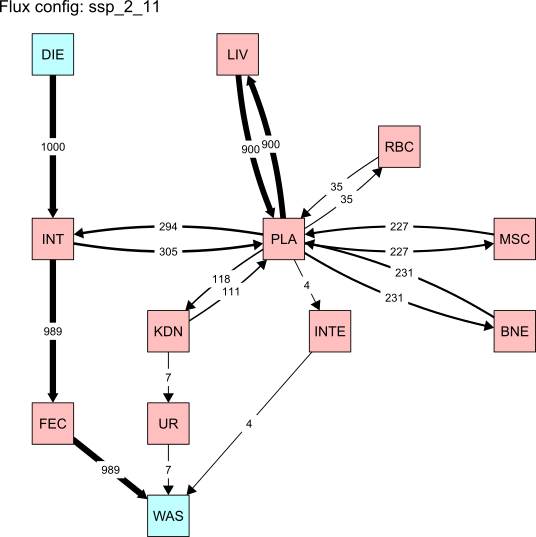


**Figure 6.** Updated flux configuration (in µg/d) for all compartments (diet (DIE), intestine (INT), plasma (PLA), erythrocytes (i.e., red blood cells; RBC), liver (LIV), muscles (MSC), bones (BNE), kidneys (KDN), hair (considered here as integument; INTE), urine (UR), feces (FEC), and waste (WAS)) from ssp_2_11, corresponding to a 700 days residence time of Zn in integument.

## Supplier – lucerne diet change with varying bone *t_1/2_*

Following the same approach as in section 3, we estimate the best residence time of Zn in the bone that could explain why it appears out of isotopic equilibrium in the rats fed for ca. 60 days on the lucerne pellets. By modulating the flux of Zn from plasma to bone and from bone to plasma, we let the residence time of Zn in bone vary between 14 d (as suggested previously in House and Wastney, 1997) up to roughly 3000 d, as assumed if Zn has the same residence time as Ca (e.g., 2000 days if 1kg Ca in bone and 500 mg/d exchanged; Del Valle et al., 2011**)** [2,7].


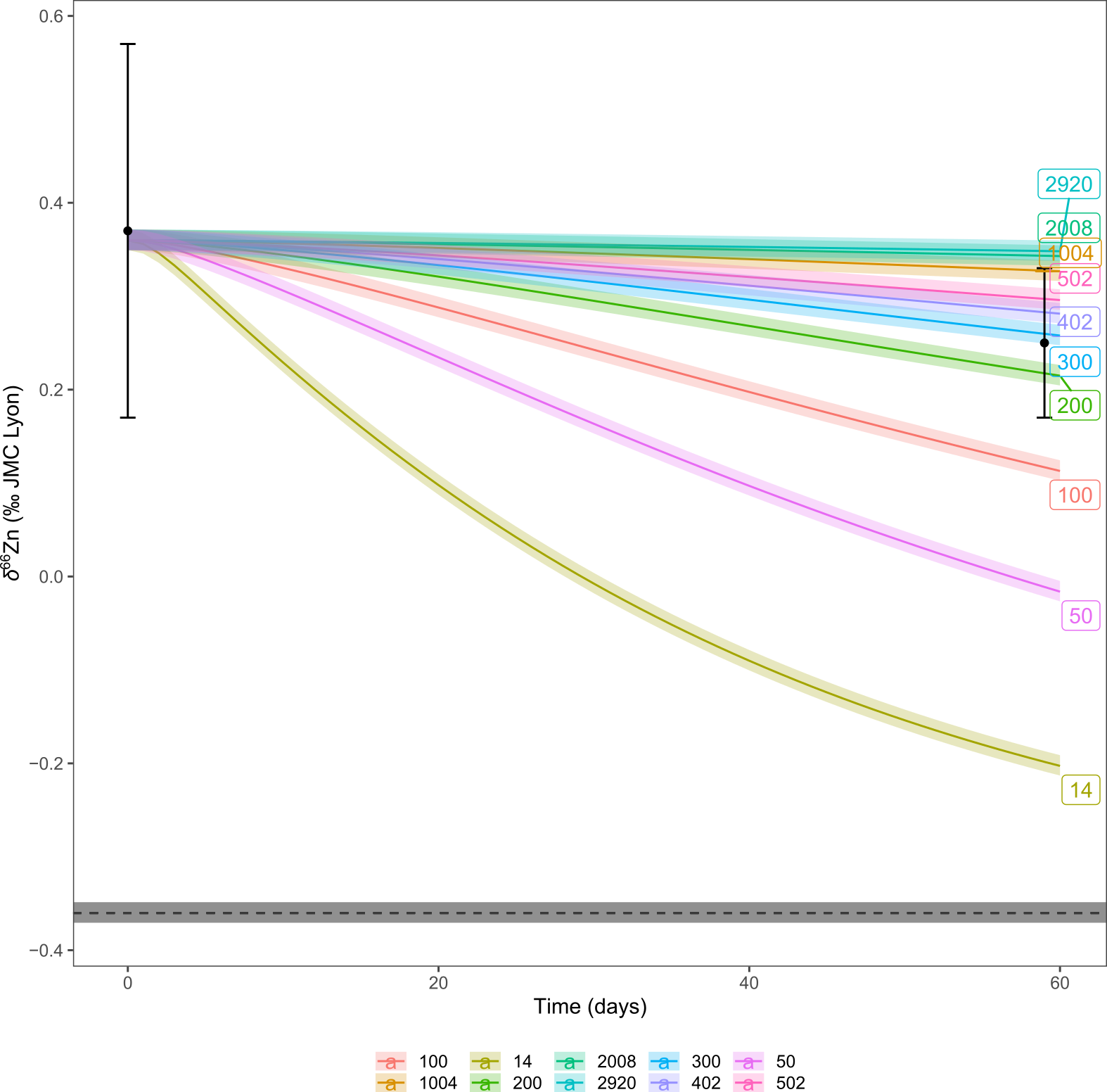


**Figure 7.** Predicted evolution of δ^66^Zn (‰ JMC-Lyon) in bone following a dietary transition from a steady-state organism at equilibrium with the supplier diet to a lucerne pellet diet, whereby different PLA-BNE fluxes (from 1.1 to 231.1 μg/day) are modeled and correspond to varying residence time (14 to 2920 days as shown in legend and on labels). The colored lines shown with the shaded area correspond to the predicted steady-state isotope composition when the organism is fully equilibrated with the second diet (lucerne pellets here) based on varying residence time. The black circles correspond to the average values of the observed δ^66^Zn values (error bars are 2SD). The dashed lines with a shaded area correspond to the predicted steady-state isotope composition when the organism is fully equilibrated with the second diet. The time axis corresponds to the time (in days) that elapsed since the start of the experiment (i.e., the introduction of the second diet). Each curve and shaded area represent the average and full extent of compositions predicted by a set of fitted parameter values but with different bone t_1/2_.

The resulting simulations suggest the best fit for bone *t_1/2_* at around 300 days, which is much higher and hardly compatible with the 14 days estimate by House and Wastney 1997 [2]. While the uncertainties on t_0_ and t_60_ bone *δ*^66^Zn values might allow for higher t_1/2_ values of up to 3000 days, our 300 days estimate seems to be lower than the residence time of Ca (2000–3000 days). We thus thereafter assume a *t_1/2_* of 300 days in bone, corresponding to a PLA-BNE and BNE-PLA fluxes of 10.7 µg/d (**Figure 8**), and used for subsequent modelling.


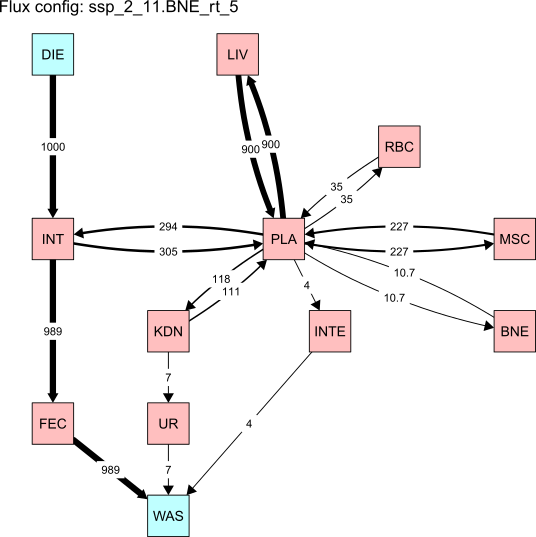


**Figure 8.** Updated flux configuration (in µg/d) for all compartments (diet (DIE), intestine (INT), plasma (PLA), erythrocytes (i.e., red blood cells; RBC), liver (LIV), muscles (MSC), bones (BNE), kidneys (KDN), hair (considered here as integument; INTE), urine (UR), feces (FEC), and waste (WAS)) taking in account a 300 days residence time of Zn in bone.

## References

1. Tacail T. (2023) isobxr: Stable isotope box modelling in R, version 2.0. https://cran.r-project.org/web/packages/isobxr/index.html.

2. House WA, Wastney ME. Compartmental analysis of zinc kinetics in mature male rats. *Am J Physiol-Regul Integr Comp Physiol* 1997;**273**:R1117–25.

3. Høyer H, Weismann K. The effect of oral zinc loading on the absorption of ^65^zinc in the rat. *Arch Dermatol Res* 1979;**264**:327–30.

4. Milne DB, Canfield WK, Mahalko JR, Sandstead HH. Effect of dietary zinc on whole body surface loss of zinc: Impact on estimation of zinc retention by balance method. *Am J Clin Nutr* 1983;**38**:181–6.

5. Jaouen K, Pouilloux L, Balter V, Pons M-L, Hublin J-J, Albarède F. Dynamic homeostasis modeling of Zn isotope ratios in the human body. *Metallomics* 2019;**11**:1049–59.

6. Moore RET, Rehkämper M, Maret W, Larner F. Assessment of coupled Zn concentration and natural stable isotope analyses of urine as a novel probe of Zn status†. *Metallomics* 2019;**11**:1506–17.

7. Del Valle HB, Taylor CL, Yaktine AL, Ross AC eds. *Dietary Reference Intakes for Calcium and Vitamin D*. National Academies Press, 2011.

# ****Supplementary Material-3: Dietary switch****


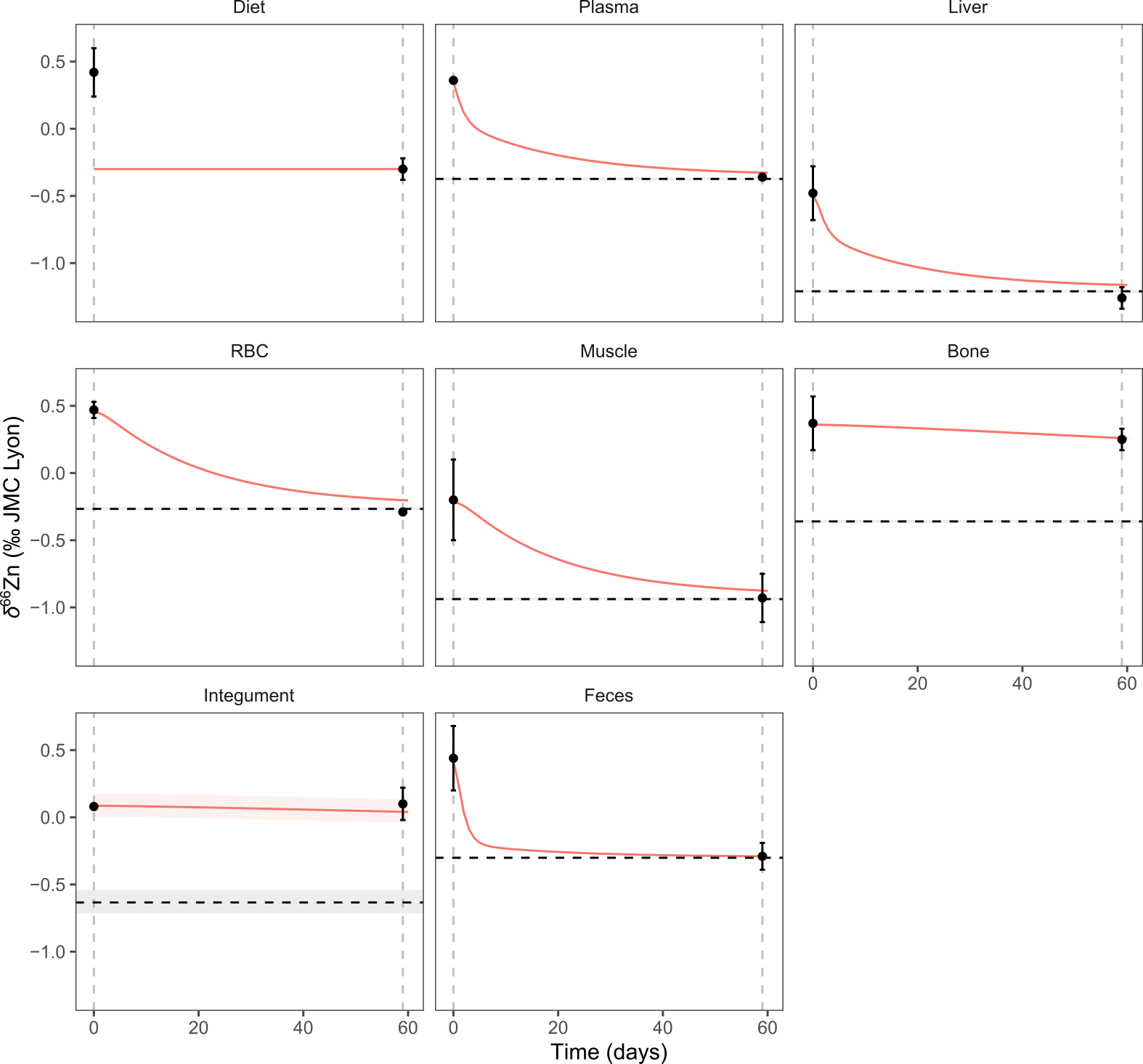


**Figure S1.** Predicted evolution of *δ*^66^Zn (‰ JMC-Lyon) in each box following a dietary transition from a steady-state organism at equilibrium with the supplier diet to a pelleted lucerne diet. The dashed lines with a shaded area correspond to the predicted steady-state isotope composition when the organism is fully equilibrated with the second diet (lucerne pellets here). The black circles correspond to the average values of the observed *δ*^66^Zn values (error bars are 2SD). The time axis corresponds to the time (in days) that elapsed since the start of the experiment (i.e., the introduction of the second diet). Each curve and shaded area represent the average and full extent of compositions predicted by the sets of fitted parameter values determined for each flux configuration (see fit_4 described in ***Supplementary Material-2*** and Figure 8 of ***Supplementary Material-2***).


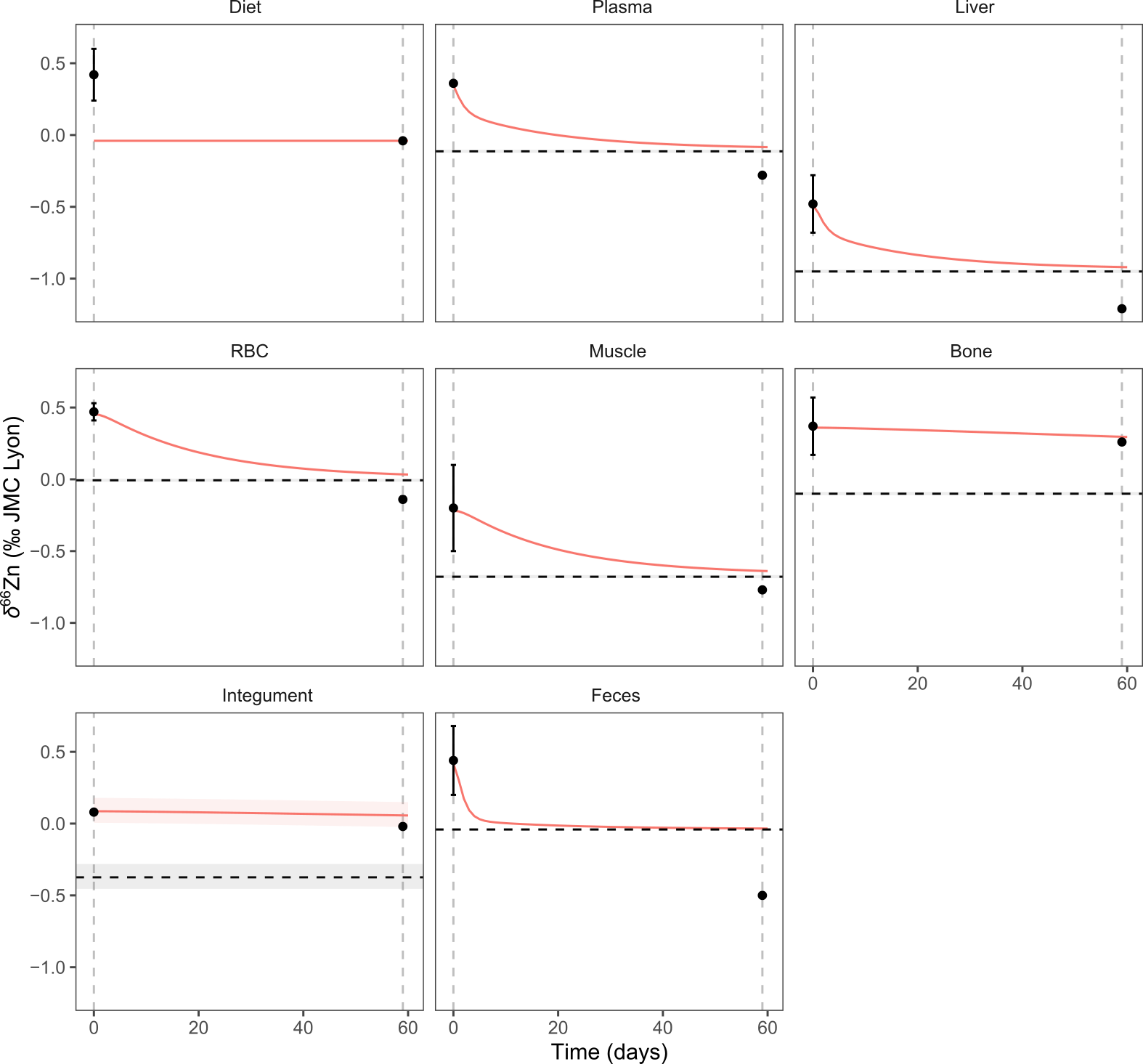


**Figure S2.** Predicted evolution of *δ*^66^Zn (‰ JMC-Lyon) in each box following a dietary transition from a steady-state organism at equilibrium with the supplier diet to a pelleted insect meal diet. The dashed lines with a shaded area correspond to the predicted steady-state isotope composition when the organism is fully equilibrated with the second diet (pelleted insect meal here). The black circles correspond to the average values of the observed *δ*^66^Zn values (error bars are 2SD). The time axis corresponds to the time (in days) that elapsed since the start of the experiment (i.e., the introduction of the second diet). Each curve and shaded area represent the average and full extent of compositions predicted by the sets of fitted parameter values determined for each flux configuration (see fit_4 described in ***Supplementary Material-2*** and Figure 8 of ***Supplementary Material-2***).


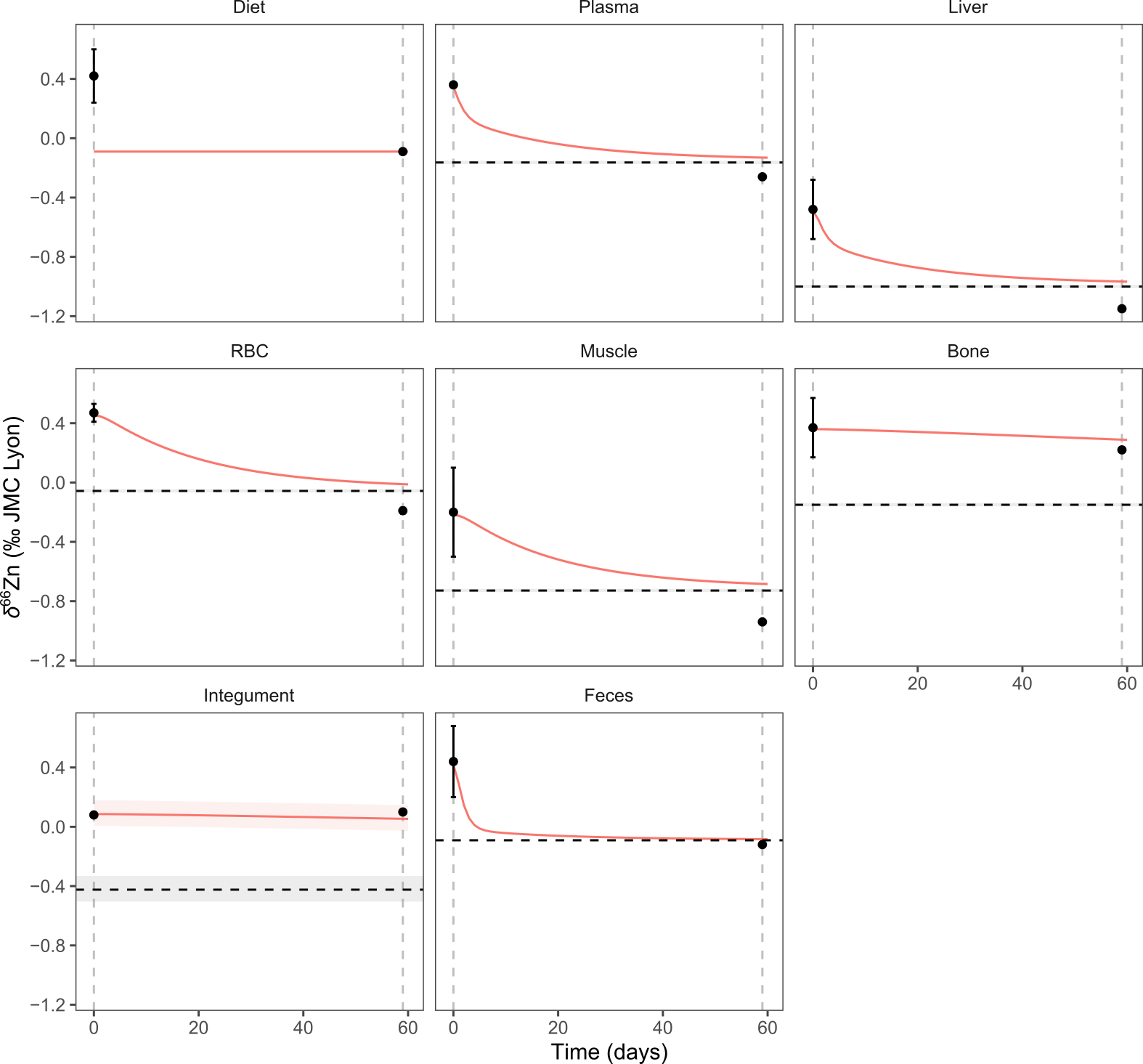


**Figure S3.** Predicted evolution of *δ*^66^Zn (‰ JMC-Lyon) in each box following a dietary transition from a steady-state organism at equilibrium with the supplier diet to a pelleted animal meal diet. The dashed lines with a shaded area correspond to the predicted steady-state isotope composition when the organism is fully equilibrated with the second diet (pelleted animal meal here). The black circles correspond to the average values of the observed *δ*^66^Zn values (error bars are 2SD). The time axis corresponds to the time (in days) that elapsed since the start of the experiment (i.e., the introduction of the second diet). Each curve and shaded area represent the average and full extent of compositions predicted by the sets of fitted parameter values determined for each flux configuration (see fit_4 described in ***Supplementary Material-2*** and Figure 8 of ***Supplementary Material-2***).


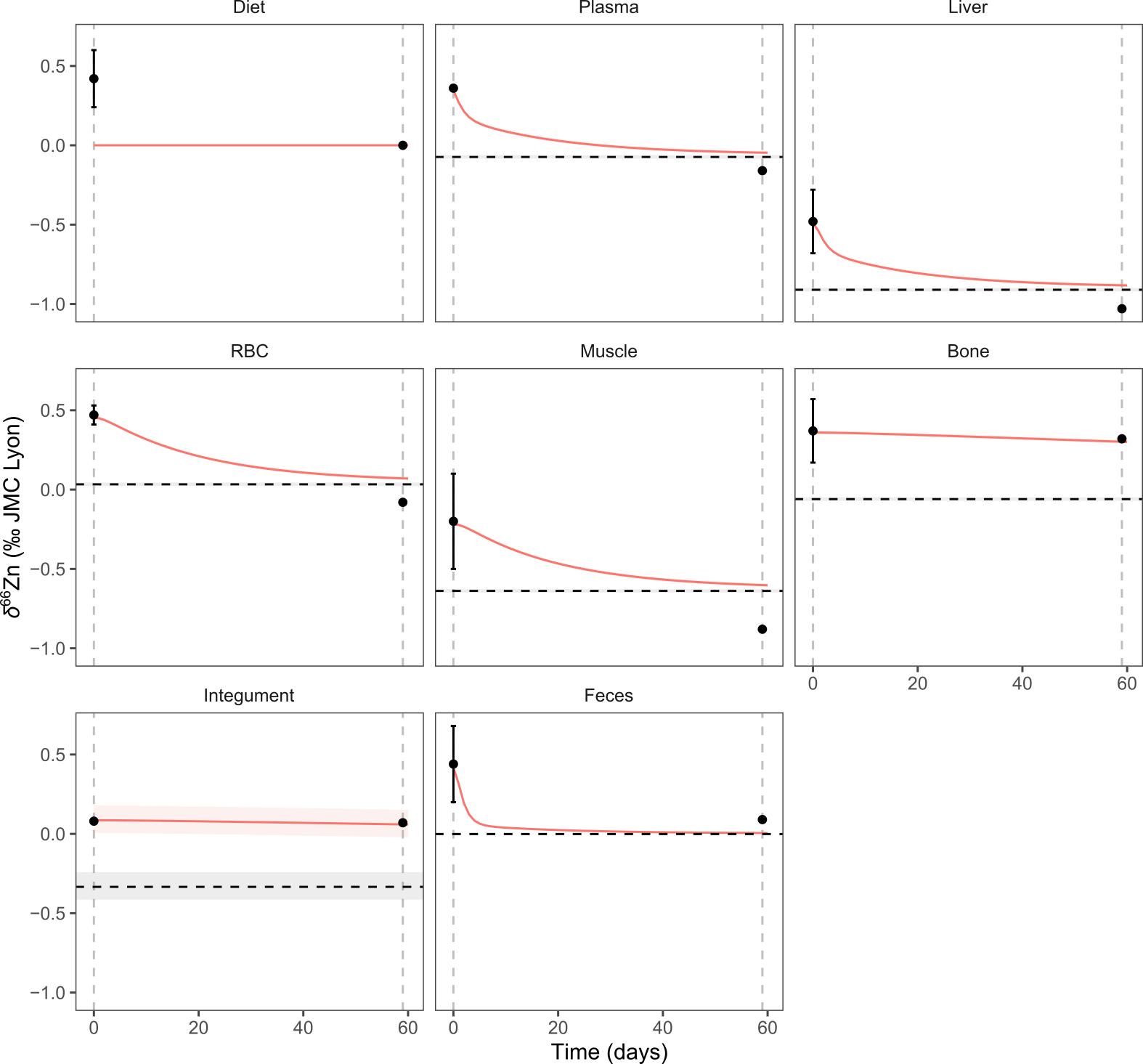


**Figure S4**. Predicted evolution of *δ*^66^Zn (‰ JMC-Lyon) in each box following a dietary transition from a steady-state organism at equilibrium with the supplier diet to a pelleted animal meal diet with a bone-meal supplement totaling 14% of the feed’s weight. The dashed lines with a shaded area correspond to the predicted steady-state isotope composition when the organism is fully equilibrated with the second diet (pelleted 14% “bone” meal here). The black circles correspond to the average values of the observed *δ*^66^Zn values (error bars are 2SD). The time axis corresponds to the time (in days) that elapsed since the start of the experiment (i.e., the introduction of the second diet). Each curve and shaded area represent the average and full extent of compositions predicted by the sets of fitted parameter values determined for each flux configuration (see fit_4 described in ***Supplementary Material-2*** and Figure 8 of ***Supplementary Material-2***).


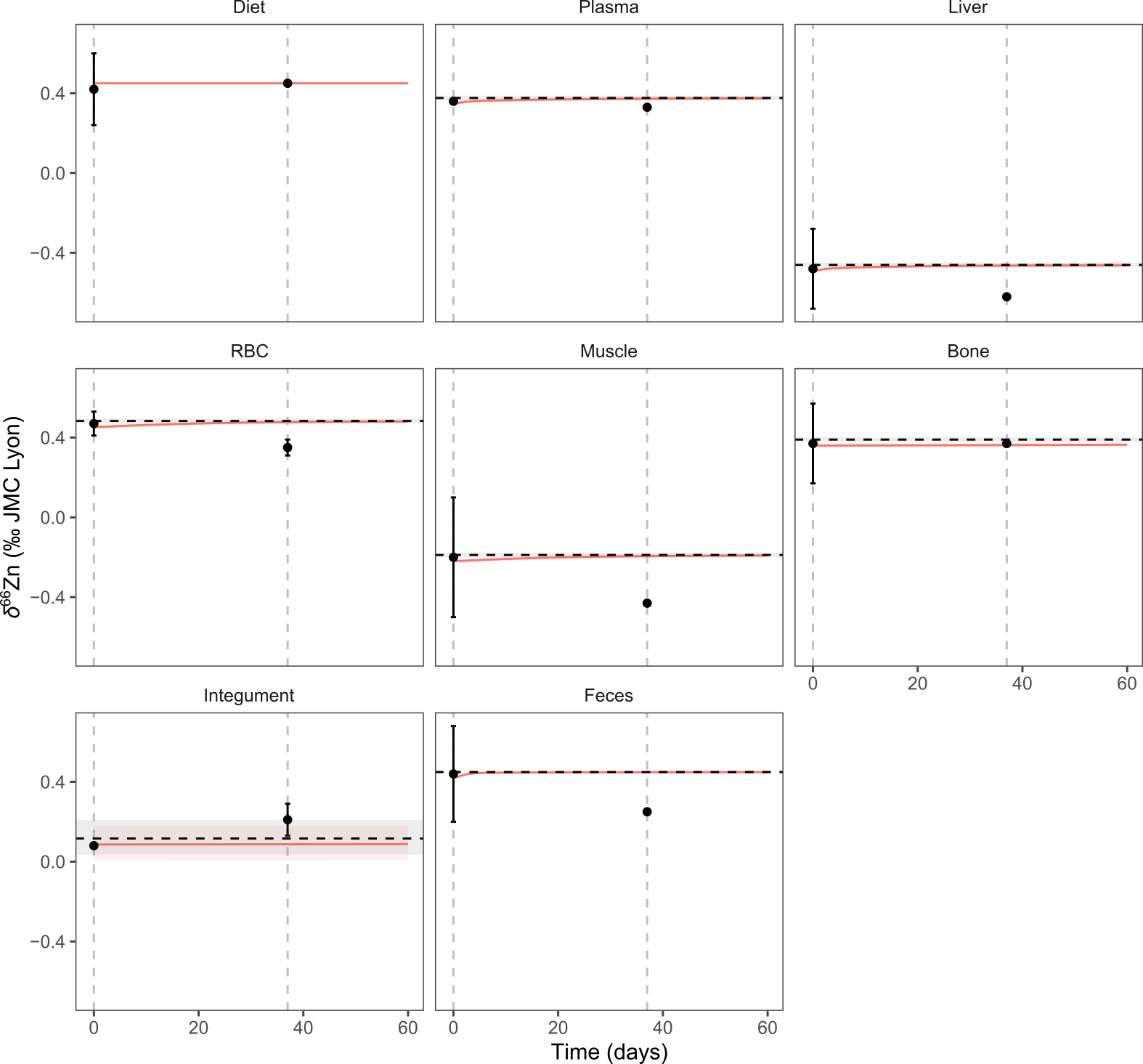


**Figure S5.** Predicted evolution of *δ*^66^Zn (‰ JMC-Lyon) in each box following a dietary transition from a steady-state organism at equilibrium with the supplier diet to a vegetable mix diet. The dashed lines with a shaded area correspond to the predicted steady-state isotope composition when the organism is fully equilibrated with the second diet (vegetable mix diet here). The black circles correspond to the average values of the observed *δ*^66^Zn values (error bars are 2SD). The time axis corresponds to the time (in days) that elapsed since the start of the experiment (i.e., the introduction of the second diet). Each curve and shaded area represent the average and full extent of compositions predicted by the sets of fitted parameter values determined for each flux configuration (see fit_4 described in ***Supplementary Material-2*** and Figure 8 of ***Supplementary Material-2***).


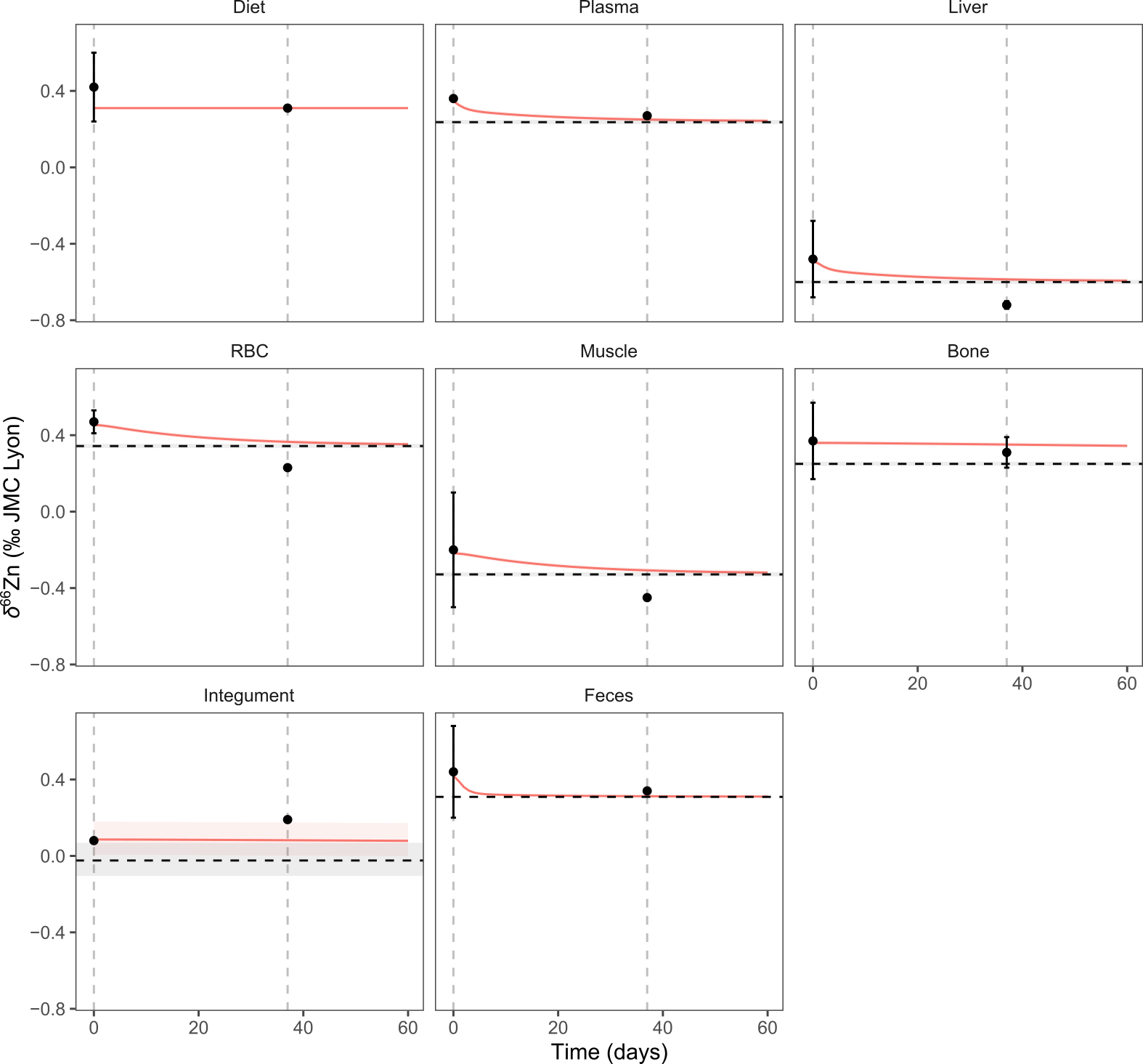


**Figure S6.** Predicted evolution of *δ*^66^Zn (‰ JMC-Lyon) in each box following a dietary transition from a steady-state organism at equilibrium with the supplier diet to a day-old-chicks diet. The dashed lines with a shaded area correspond to the predicted steady-state isotope composition when the organism is fully equilibrated with the second diet (day-old-chicks diet here). The black circles correspond to the average values of the observed *δ*^66^Zn values (error bars are 2SD). The time axis corresponds to the time (in days) that elapsed since the start of the experiment (i.e., the introduction of the second diet). Each curve and shaded area represent the average and full extent of compositions predicted by the sets of fitted parameter values determined for each flux configuration (see fit_4 described in ***Supplementary Material-2*** and Figure 8 of ***Supplementary Material-2***).


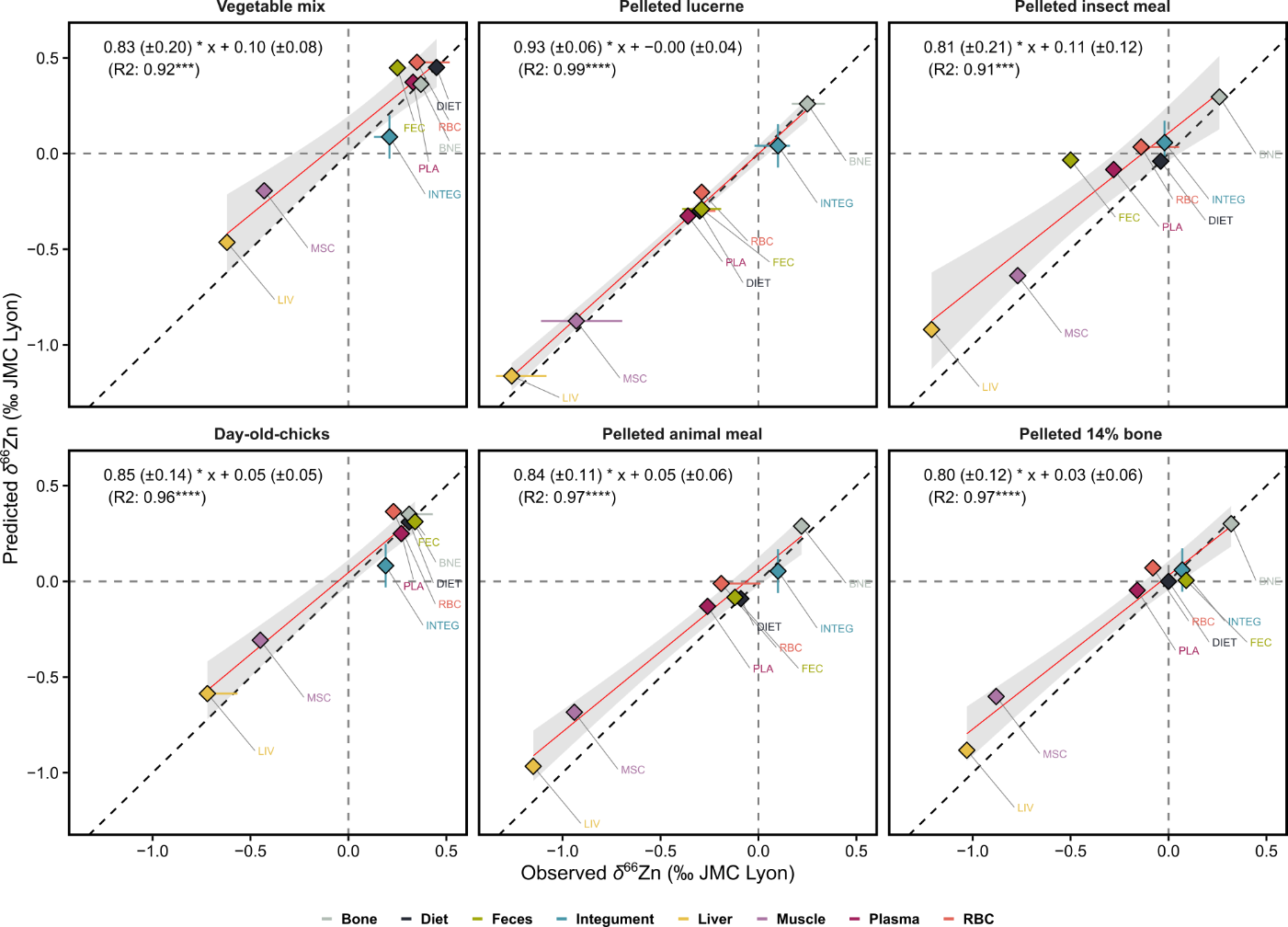


**Figure S7.** Fitness of predictions between predicted and observed *δ*^66^Zn (‰ JMC-Lyon) for each diet and according tissue and excreta samples for the respective feeding experiments, whereby the dashed lines represent a 1:1 regression between the two while the blue lines represent the actual regression. The shaded areas correspond to the 95% confidence interval of their respective regression line.
